# Supplementary material for: Estimating causal effects of physical disability and number of comorbid chronic diseases on risk of depressive symptoms in an elderly Chinese population: a machine learning analysis of cross-sectional baseline data from the China longitudinal ageing social survey
Source: BMJ Open. 2023 Jul 5;13(7):e069298. doi: 10.1136/bmjopen-2022-069298 (PMC10335586; doi:10.1136/bmjopen-2022-069298)
Supplement: Author's manuscript [file bmjopen-2022-069298.draft_revisions.pdf]

BMJ Open

BMJ Open is committed to open peer review. As part of this commitment we make the peer review history of every article we publish publicly available.

When an article is published we post the peer reviewers' comments and the authors' responses online. We also post the versions of the paper that were used during peer review. These are the versions that the peer review comments apply to.

The versions of the paper that follow are the versions that were submitted during the peer review process. They are not the versions of record or the final published versions. They should not be cited or distributed as the published version of this manuscript.

BMJ Open is an open access journal and the full, final, typeset and author-corrected version of record of the manuscript is available on our site with no access controls, subscription charges or pay-per-view fees (http://bmjopen.bmj.com).

If you have any questions on BMJ Open's open peer review process please email info.bmjopen@bmj.com

BMJ Open

Estimating causal effects of physical disability and number of comorbid chronic diseases on depressive symptom risk in an elderly Chinese population: A machine learning study

Journal:	*BMJ Open*
Manuscript ID	bmjopen-2022-069298
Article Type:	Original research
Date Submitted by the Author:	17-Oct-2022
Complete List of Authors:	Wang, Zhenjie; Peking University<br>Yang, Hanmo; Harvard University<br>Sun, Chenxi; Peking University; Peking University<br>Hong, Shenda; Peking University; Health Science Center of Peking University
Keywords:	Health informatics < BIOTECHNOLOGY & BIOINFORMATICS, MENTAL HEALTH, Depression & mood disorders < PSYCHIATRY

SCHOLARONE™
Manuscripts

I, the Submitting Author has the right to grant and does grant on behalf of all authors of the Work (as defined in the below author licence), an exclusive licence and/or a non-exclusive licence for contributions from authors who are: i) UK Crown employees; ii) where BMJ has agreed a CC-BY licence shall apply, and/or iii) in accordance with the terms applicable for US Federal Government officers or employees acting as part of their official duties; on a worldwide, perpetual, irrevocable, royalty-free basis to BMJ Publishing Group Ltd ("BMJ") its licensees and where the relevant Journal is co-owned by BMJ to the co-owners of the Journal, to publish the Work in this journal and any other BMJ products and to exploit all rights, as set out in our licence.

The Submitting Author accepts and understands that any supply made under these terms is made by BMJ to the Submitting Author unless you are acting as an employee on behalf of your employer or a postgraduate student of an affiliated institution which is paying any applicable article publishing charge ("APC") for Open Access articles. Where the Submitting Author wishes to make the Work available on an Open Access basis (and intends to pay the relevant APC), the terms of reuse of such Open Access shall be governed by a Creative Commons licence – details of these licences and which Creative Commons licence will apply to this Work are set out in our licence referred to above.

Other than as permitted in any relevant BMJ Author's Self Archiving Policies, I confirm this Work has not been accepted for publication elsewhere, is not being considered for publication elsewhere and does not duplicate material already published. I confirm all authors consent to publication of this Work and authorise the granting of this licence.

For peer review only

Estimating causal effects of physical disability and number of comorbid chronic diseases on depressive symptom risk in an elderly Chinese population: A machine learning study

Zhenjie Wang,[1*] Hanmo Yang,[4] Chenxi Sun,[5,6], Shenda Hong,[2,3*]

[1] Institute of Population Research, Peking University, Beijing 100871, People's Republic of China

[2] National Institute of Health Data Science, Peking University, Beijing 100191, People's Republic of China

[3] Institute of Medical Technology, Health Science Center of Peking University, Beijing 100191, People's Republic of China

[4] T.H. Chan School of Public Health, Harvard University, Boston 02115, United States of America

[5] School of Electronics Engineering and Computer Science, Peking University, Beijing, People's Republic of China

[6] Key Laboratory of Machine Perception (Ministry of Education), Peking University, Beijing, People's Republic of China

[*] **Correspondence:** Zhenjie Wang, Institute of Population Research, Peking University, No. 5 Yiheyuan Road, Haidian District, Beijing 100871, People's Republic of China; E-mail: zhenjie.wang@pku.edu.cn. Shenda Hong, National Institute of Health Data Science, Peking University, Beijing 100191, People's Republic of China；E-mail: hongshenda@pku.edu.cn

ABSTRACT

Objective This study aimed to explore the causal effects of physical disability and number of comorbid chronic diseases on depressive symptoms in an elderly Chinese population.

Design, setting and analysis Data were obtained from the China Longitudinal Ageing Social Survey (CLASS), which is a stratified, multistage, probabilistic sampling survey conducted in 2014 that covers 28 of 31 provincial areas in China. The causal effects of physical disability and number of comorbid chronic diseases on depressive symptoms were analyzed by using the conditional average treatment effect (CATE) method.

Outcome The outcome for this study was the assessment of the causal effects of physical disability and number of comorbid chronic diseases on depressive symptoms.

Participants A total sample of 7496 subjects aged 60 years or older who answered the questions on depressive symptoms and other independent variables of interest in this study were included.

Results In the present study, the sample comprised 7496 subjects aged 60 years or older who answered the questions on depressive symptoms and other independent variables of interest. Physical disability and the number of comorbid chronic diseases had causal effects on depressive symptom (DS). Among the subjects who had one and more functional limitations, one chronic disease and two or more chronic diseases, the possibility of DS increased by 22%, 13%, and 20%, respectively.

Conclusion: The present study provides evidence that one and more functional limitations affect the occurrence of depressive symptoms among elderly people. This is helpful for planning a possible avenue for preventive measures and for improving knowledge on how to

For peer review only

treat depressive symptoms. The findings of our study are valuable for the development of prevention programs to identify elderly individuals who have physical disabilities or a number of comorbid chronic diseases and for early intervention.

Keywords: Health informatics, mental health, causal effects, older population, Chinese

For peer review only

Strengths and limitations of this study

- The current study used a large, representative population-based sample covering 28 of 31 provincial areas in China.
- The study estimated causal effects by using the conditional average treatment effect (CATE) method.
- The findings of this cross-sectional study established a cause-and-effect relationship by using a machine learning algorithm.

INTRODUCTION

Currently, depressive symptoms (DS) are one of the most prevalent mental disorders worldwide.[1] The characteristics of DS include symptoms of sadness, depressed mood, and loss of interest.[1] People who have DS are usually associated with having low quality of life, cancer, chronic diseases, suicide, and so on.[2-5] DS can also place a heavy burden on families, communities and health services worldwide.[6, 7] The prevalence of DS varies widely across the world, from 1% to 16% among middle-aged and elderly populations.[8-10] In China, the prevalence of DS has increased rapidly from 3.9% to 17% among elderly people in the past two decades.[11-13]

Many studies have provided evidence that several demographic variables are associated with DS risk,[14] although the associations are inconsistent.[10, 13] An inverse or U-shaped association between age and DS has been found in many studies.[15-17] Demographic status and education were associated with DS risk.[18-20] However, some studies reported no association among demographic status, education and DS.[14,17,21]

Very few epidemiologic studies have provided evidence on the causal effects of demographic factors on DS risk in the elderly Chinese population. Therefore, we explored the causal effects of physical disability and number of comorbid chronic diseases on depressive symptom risk and estimated the conditional average treatment effect (CATE) of physical disability and number of comorbid chronic diseases on depressive symptoms using data from the Chinese Longitudinal Ageing Social Survey (CLASS) by using machine learning.

METHODS AND MATERIALS

For peer review only

Data source

In the current study, we drew the sample from the Chinese Longitudinal Ageing Social Survey (CLASS) in 2014. The CLASS was collected by the National Survey Research Center, Renmin University. A stratified, multistage, probabilistic sampling method was employed to select a nationally representative sample, covering 28 of 31 provincial areas in China. A total of 11,511 older adults were surveyed. In the present study, the sample comprised 7496 subjects aged 60 years or older who answered the questions on depressive symptoms and other independent variables of interest. All the participants were interviewed face-to-face by trained interviewers.

Measurement of Variables

Depressive symptoms

Depressive symptoms were assessed by using a 9-item Center for Epidemiological Studies Depression Scale (CES-D), including three items assessing positive feelings, two items assessing negative emotions, two items assessing somatic symptoms, and two items assessing sense of marginalization. The 9-item CES-D is reliable and valid for detecting nonpsychotic mental disorders among older Chinese adults.[24] Each item had a score of 0 (rarely or none of the time), 1 (some of the time), or 2 (most of the time), with the total score ranging from 0 to 18. By reversing the coding of the positive effect items, a higher score indicates a higher level of depressive symptoms. For the current study, on a 9-item scale, the total possible score was 18 (9 items multiplied by 2, the highest response). The standardized cut score of 4.8 for the 9-item form of the CES-D by using Kohout's formula. [25] In this study, the internal Cronbach's

For peer review only

alpha for the nine items was 0.75.

Sociodemographic Characteristics

Demographic characteristics included gender (male, female), age, marital status (married, widowed/divorced/unmarried), ethnicity (Han, others), residence (rural, urban), education level (junior high school and above, primary school, never attended school), and living arrangements (lives alone, lives with others). A ten-item version of the activities of daily living (ADLs) was used to assess physical disability.[26] The number of comorbid chronic diseases, including any health problems such as hypertension, diabetes, heart disease, renal disease, liver disease, stroke, tuberculosis, arthritis, and respiratory diseases, were categorized as “0”, “1” and “≥2”.

Estimating Causal Effects

Causal analysis aims to determine the causal relationships between variables (treatments) and outcomes. Causal relationships are essentially different from correlations in that causal relationships mainly solve the problem of "why". In this work, we used Causal ML,[27] an open source Python software provided by Uber, for causal analysis methods using machine learning algorithms based on recent research. Causal ML provides a standard interface that allows users to estimate the average treatment effect (ATE) from experimental or observational data.

To estimate the causal relationships between the variable of interest (known as treatment indicator E) and the outcome Y, we performed a two-step analysis: (1) we built a machine

For peer review only - http://bmjopen.bmj.com/site/about/guidelines.xhtml

learning model and (2) estimated the ATE based on Causal ML. First, we built a random forest classifier (RFC) for predicting DS using all the variables except for the treatment indicator (denoted as X). [28] The RFC contains 100 decision trees and uses the Gini purity index to judge the segmentation criteria of a tree node. The maximum depth of a single tree is set to unlimited so that each tree is segmented until all the leaf nodes of the tree model have only one sample. The RFC output 1 if the individual had DS and output 0 otherwise. The RFC model was built using a scikit-learn machine learning framework. [29]

Then, we set the above trained RFC as the base learner for Causal ML and estimated the treatment effect based on the algorithm described herein.[30] The algorithm used a single base learner from the machine learning model while having the treatment indicator as a feature. It consisted of two stages. In the first stage, the algorithm estimated the average outcomes for treatments using the trained RFC model, calculated as $\mu(x) = E(Y|X = x, E = e)$. In the second stage, the ATE was calculated as $\tau(x) = \mu(x, E = 1) - \mu(x, E = 0)$. In addition, we also used SHAPley Additive exPlanations (SHAP), a game theoretic approach, to explain the output of the causal analysis results modeled by the machine learning models. [31] We analyzed data by using Python 3.7.14 based on anaconda3 development platform, causalml version 0.10.0, sklearn version 0.23.2, pandas version 1.0.1, numpy version 1.21.0.

Patient and public involvement

The interviewer-administered questionnaire was prepared in local language (Chinese). Consent was received from each participant before the data collection and the data collectors were trained to provide any information or clarification at any time of the interview. Only

For peer review only

participants who wish to continue the study after informed consent were included in the study. In the current study, no patients were involved.

RESULTS

Descriptive Statistics

Select characteristics of subjects with/without depressive symptoms (DS) are summarized in Table 1. The overall average Center for Epidemiological Studies Depression Scale (CES-D) score was 4.56 (standard deviation: 3.56). The prevalence of DS was 43% among the Chinese population aged over 60 years. In the current study, male subjects, urban residents, people living with others, and people of Han ethnicity accounted for the majority proportion of elderly Chinese individuals with DS.

Table 1 Subjects' characteristics by their depressive symptoms status according to the 9-item Center for Epidemiological Studies Depression Scale (CES-D) among elder population in China

Characteristics	Without depressive symptom	With depressive symptom	*P*
Age groups (years)			
60-64	1603 (37.2)	1068 (33.5)	< 0.001
65-69	988 (22.9)	704 (22.1)	
70-74	766 (17.8)	547 (17.2)	
75-79	530 (12.3)	471 (14.8)	
80+	422 (9.8)	397 (12.5)	
Residence			
Rural	1207 (28.0)	1315 (41.3)	< 0.001
Urban	3102 (72.0)	1872 (58.8)	
Gender			
Male	2427 (56.3)	1627 (51.1)	< 0.001
Female	1882 (43.7)	1563 (48.9)	
Marital status			
Married	3316 (77.0)	2038 (63.9)	< 0.001
Widowed/divorced/ Unmarried	993 (23.0)	1149 (36.1)	
Education level			
Never attended school	651 (15.1)	816 (25.6)	< 0.001
Primary school	1409 (32.7)	1266 (39.7)	

Junior High school and above	2249 (52.2)	1105 (34.7)	
Ethnicity			
Han	4074 (94.5)	2990 (93.8)	0.19
Others	235 (5.5)	197 (6.2)	
Living arrangement			
Live with others	3925 (91.1)	2666 (83.7)	< 0.001
Live alone	384 (8.9)	521 (16.3)	
Physical disability			
No function problems	3988 (92.6)	2531 (79.4)	< 0.001
One and more functioning limitations	321 (7.4)	656 (20.6)	
Wealth quantile			
Q1 (lowest)	691 (16.0)	925 (29.0)	< 0.001
Q2	694 (16.1)	717 (22.5)	
Q3	1066 (24.7)	705 (22.1)	
Q4	1002 (23.3)	520 (16.3)	
Q5 (highest)	856 (19.9)	320 (10.1)	
Number of comorbid chronic disease			
0	1463 (34.0)	551 (17.3)	< 0.001
1	1307 (30.3)	917 (28.8)	
≥2	1539 (35.7)	1719 (53.9)	

For peer review only

Estimated Causal Effects

The estimated causal effects of physical disability and chronic diseases on depressive symptoms are presented in Table 2. Physical disability and the number of comorbid chronic diseases had causal effects on DS. Among the subjects who had one and more functional limitation, one chronic disease, and two or more chronic diseases, the possibility of DS increased by 22%, 13%, and 20%, respectively.

Table 2 Estimate causal mechanisms effect of physical disability, chronic diseases on depressive symptoms

	Reference		Causal mechanisms effect (95% CI)
Physical disability *	No function problems	One and more functioning limitations	0.22 (0.19, 0.24)
Number of comorbid chronic disease †	0	1	0.13 (0.10, 0.15)
		≥2	0.20 (0.18, 0.22)

* under consideration causal mechanisms effect of age group, gender, residence, marital status, education level, ethnicity, wealth quantile, wealth quantile and number of comorbid chronic disease.

† under consideration causal mechanisms effect of age group, gender, residence, marital status, education level, ethnicity, wealth quantile, wealth quantile and physical disability.

In descending order of importance the effects of the mechanism direction of physical disability and number of comorbid chronic diseases on DS are presented in Figure 1-2. In descending order of importance among subjects who had one and more functional limitations, the variables are as follows: education level, age group, number of comorbid chronic diseases, wealth quantile, marital status, living arrangement, residence, gender and ethnicity. In descending order of importance among subjects who had only one chronic disease, the variables are as follows: wealth quantile, age group, education level, residence, gender, marital status, physical disability, living arrangement and ethnicity. In descending order of importance among subjects who had one and more functional limitations, the variables are as follows: wealth quantile, age group, residence, education level, gender, marital status, living arrangement, physical disability and ethnicity. Age group, residence, gender, and physical disability contributed to DS risk among subjects who had one or more chronic diseases.

DISCUSSION

Overall, physical disability and the number of comorbid chronic diseases had causal effects on depressive symptom (DS) risk. Subjects who had one and more functional limitations had a 22% increased possibility of DS risk. There was an increased 13% and 20% possibility of DS risk among the subjects who had one chronic disease and two or more chronic diseases, respectively.

In the current study, we found causal effects of physical disability and comorbid chronic diseases on DS. A previous study suggested that some health variables, such as poor self-rated health and disability, were associated with DS, indicating that health status tends to have similar effects on mental health regardless of social context.[32] Physical disability is

For peer review only

considered the inability or diminished capacity to perform basic self-care activities. Physical disability undermines an individual's ability to interact with the physical and social environment, hence increasing the risk of DS.[33] Increased disability in activities of daily living (ADLs) and instrumental activities of daily living (IADLs) have been proven to be connected with depressive symptoms.[34,35] Failure to carry out ADLs has been positively associated with depressive symptoms in elderly people, and physical disability accounts for incidences of depressive symptoms in elderly individuals.[36,37] Furthermore, that physical disability leads to increased geriatric depressive symptoms over time has been supported by longitudinal studies.[38] Given the influence of physical disability and depressive symptoms on the well-being of elderly individuals, it is urgent to find ways to address these issues. The results demonstrated that impairment of ADLs had a significant effect on depressive symptoms in elderly people, conforming with the findings of previous studies showing a relationship between activity limitations and psycho-social problems.[34,35]

The number of chronic diseases was associated with DS risk, which was consistent with previous studies. These associations could be explained by several effects, including unhealthy lifestyle, shared genetic influences, and inflammatory factors in diseases.[39] Previous studies also suggested some behavioral or biological pathways that link DS with diabetes.[30] Previous studies suggested that pain and physical disability experience could lead to a side effect on the mood of elderly individuals.[20] Another explanation is that subjects with depressive symptoms might be more sedentary than those without depressive symptoms, which could cause increased bone resorption and decreased bone formation.[40,41]

For peer review only

Strengths and limitations of this study

Our study has many strengths, including a large sample size, a population-based design, and adjustment for a wide range of socioeconomic characteristics. Another noticeable strength is that the measurement of all physical illnesses took place prior to the CES-D measurement, minimizing the risk of reverse causation. However, our study also has several limitations, which should be considered by future researchers. First, although we used machine learning to estimate causal effects of the factors on DS, the cross-sectional design of the baseline of the CLASS should be considered cautiously in future studies. We will be able to extend the current study to determine causality when longitudinal data are available. Moreover, the CLASS does not provide sufficient information on lifestyle factors (i.e., weight, height, smoking, alcohol drinking, and so on), which were suggested as risk factors for depression.[42-44]

CONCLUSION

In conclusion, in this large population-based study in an elderly Chinese population, physical disability and the number of comorbid chronic diseases increased DS risk. The present study provides evidence of the effect of one and more functional limitations on the occurrence of depressive symptoms among elderly people. This is helpful for planning a possible avenue for preventive measures and for improving knowledge on how to treat depressive symptoms. The findings of our study are valuable for the development of prevention programs to identify elderly individuals who have physical disabilities or a number of comorbid chronic diseases and for early intervention.

For peer review only

Acknowledgments We would like to thank the Institute of Gerontology and National Survey Research Center at Renmin University of China for providing the CLASS data.

Contributors Conceptualization: Z W. Methodology: S H. Software: Z W and S H. Writing: Z W. Writing—review: Z W, S H, H Y and C S. Writing—editing: H Y and C S.

Funding This work was supported by the National Social Science Fund of China (No. 20BRK020), the National Natural Science Foundation of China (No.62102008) and Economic & Social Research Council (ESRC) (No. ES/P011055/1). The funder did not participate in the research or review any details of this study; the other authors are independent of the funder.

Competing interests None declared.

Patient consent for publication Not needed.

Ethics approval All procedures performed in this study involving human participants were in accordance with the ethical standards of the institutional and/or national research committee and with the 1964 Helsinki Declaration and its later amendments or comparable ethical standards. The surveys were conducted within the legal framework governed by statistical law in China. This article does not contain any studies with animals performed by any of the authors.

Provenance and peer review Not commissioned; externally peer reviewed.

Data availability statement The datasets generated and/or analyzed during the current study are available in the China Longitudinal Ageing Social Survey (CLASS) repository,

For peer review only

http://class.ruc.edu.cn/index.php?r=index/index&hl=en.

REFERENCES

1. Whiteford HA, Ferrari AJ, Degenhardt L, *et al*. Global Burden of Mental, Neurological, and Substance Use Disorders: An Analysis from the Global Burden of Disease Study 2010. *PLoS One*. 2015;10:e0116820.
2. Dalton SO, Laursen TM, Ross L, *et al*. Risk for hospitalization with depression after a cancer diagnosis: a nationwide, population-based study of cancer patients in Denmark from 1973 to 2003. *J Clin Oncol*. 2009; 27:1440–1445.
3. Hsu YT, Liao CC, Chang SN, Y *et al*. Increased Risk of Depression in Patients with Parkinson Disease: A Nationwide Cohort Study. *Am J Geriatr Psychiatry.* 2015; 23:934–940.
4. Ayerbe L, Ayis S, Wolfe CD, *et al*. Natural history, Predictors and outcomes of depression after stroke: systematic review and meta-analysis. *Br J Psychiatry.* 2013; 202:14-21.
5. Cuijpers P, Vogelzangs N, Twisk J, *et al*. Comprehensive meta-analysis of excess mortality in depression in the general community versus patients with specific illnesses. *Am J Psychiatry.* 2014; 171: 453–462.
6. World Health Organization. Depression–a global public health concern. 2012, http://hesp-news.org/2012/10/05/depression-a-global public-health-concern/ (accessed: 2018-11-29).
7. Walker ER, McGee RE, Druss BG. Mortality in mental disorders and global disease burden implications: a systematic review and meta-analysis. *JAMA Psychiatry.* 2015;

For peer review only

72:334–341.

8. Wilhelm K, Mitchell P, Slade T, *et al*. Prevalence and correlates of DSM-IV major depression in an Australian national survey. *J Affect Disord.* 2003; 75:155–162.
9. Waraich P, Goldner E M, Somers J M, *et al*. Prevalence and incidence studies of mood disorders: a systematic review of the literature. *Can J Psychiatry*. 2004; 49:124–138.
10. Djernes JK. Prevalence and predictors of depression in populations of elderly: a review. *Acta Psychiatr Scand.* 2006;113:372–387.
11. Chen R, Copeland J R, Wei L. A meta-analysis of epidemiological studies in depression of older people in the People's Republic of China. *Int J Geriatr Psychiatry.* 1999; 14:821–830.
12. Li D, Zhang DJ, Shao JJ, *et al*. A meta-analysis of the prevalence of depressive symptoms in Chinese older adults. *Arch Gerontol Geriatr*. 2014; 58:1–9.
13. Zhang L, Xu Y, Nie H, *et al*. The prevalence of depressive symptoms among the older in China: a meta-analysis. *Int J Geriatr Psychiatry.* 2012; 27:900–906.
14. Gao S, Jin Y, Unverzagt FW, *et al*. Correlates of depressive symptoms in rural elderly Chinese. *Int J Geriatr Psychiatry.* 2009; 24:1358–1366.
15. Kessler RC, Birnbaum HG, Shahly V, *et al*. Age differences in the prevalence and co-morbidity of DSM-IV major depressive episodes: results from the WHO World Mental Health Survey Initiative. *Depress Anxiety.* 2010; 27:351–364.
16. Pan A, Franco OH, Wang YF, *et al*. Prevalence and geographic disparity of depressive symptoms among middle-aged and elderly in China. *J Affect Disord.* 2008;105:167–175.
17. Yunming L, Changsheng C, Haibo T, *et al*. Prevalence and risk factors for depression in

For peer review only

older people in Xi'an China: a community-based study. *Int J Geriatr Psychiatry.* 2012; 27(1):31-9.

18. Lorant V, Croux C, Weich S, *et al*. Depression and socio-economic risk factors: 7-year longitudinal population study. *Br J Psychiatry*. 2007; 190:293–298.
19. Van d V, Bracke P, Levecque K. Gender differences in depression in 23 European countries. Cross-national variation in the gender gap in depression. *Soc Sci Med*. 2010; 71:305–313.
20. Zhou X, Bi B, Zheng L, *et al*. The prevalence and risk factors for depression symptoms in a rural Chinese sample population. *PLoS One.* 2014; 9:e99692.
21. van der Wurff F B, Beekman A T, *et al*. Prevalence and risk-factors for depression in elderly Turkish and Moroccan migrants in the Netherlands. *J Affect Disord.* 2004; 83:33–41.
22. Beals J, Belcourt-Dittloff A, Garroutte EM, *et al*. Trauma and conditional risk of posttraumatic stress disorder in two American Indian reservation communities. *Soc Psychiatry Psychiatr Epidemiol.* 2013; 48(6):895–905.
23. Roh S, Brown-Rice KA, Lee KH, *et al*. Stressors, coping resources, and depressive symptoms among rural American Indian older adults. *Soc Work Public Health*. 2015; 30(4):345–359.
24. Silverstein M, Cong Z, Li S. Intergenerational transfers and living arrangements of older people in rural China: consequences for psychological wellbeing. *J. Gerontol*. 2006; 61:S256–S266.
25. Kohout FJ, Berkman LF, Evans DA, Cornoni-Huntley J. Two shorter forms of the CES-D

For peer review only

depression symptoms index. *J Aging Health.* 1993;5(2):179–193.

26. Feng Q, Zhen Z, Gu D, *et al*. Trends in ADL and IADL disability in community-dwelling older adults in Shanghai, China, 1998–2008. *J Gerontol B Psychol Sci Soc Sci.* 2013; 68: 476–485.

27. Chen H, Harinen T, Lee JY, *et al*. Causalml: python package for causal machine learning. *arXiv.* (2020).

28. Breiman L ."Random Forests." *Machine Learning*. 2001; 45(1), 5-32.

29. Pedregosa F, Varoquaux G, Gramfort A, *et al*. Scikit-learn: machine learning in python. Scikit-learn: Machine learning in Python. *Journal of machine Learning research*. 2012;12: 2825-2830.

30. Künzel Sren R, Sekhon JS, Bickel PJ, Yu B. Meta-learners for estimating heterogeneous treatment effects using machine learning. *Proc Natl Acad Sci U S A.* 2017; 116: 4156-4165.

31. Lundberg S, Lee SI. A Unified Approach to Interpreting Model Predictions. In Proceedings of the 31st international conference on neural information processing system. 2017; 4768-4777.

32. Kino S, Jang SN, Kawachi I. Healthy but unhappy? Cross-national comparative analysis of depressive symptoms in Japanese vs. Korean elders. *Arch Gerontol Geriatr.* 2021; 95:104426.

33. Bruce ML. Depression and disability in late life: directions for future research. *Am J Geriatr Psychiatry*. 2001;.9(2):102-12.

34. Bowen ME, Ruch A. Depressive Symptoms and Disability Risk Among Older White and

For peer review only

Latino Adults by Nativity Status. *J Aging Health*. 2015; 27(7):1286-305.

35. Hybels CF, Pieper CF, Blazer DG. The complex relationship between depressive symptoms and functional limitations in community-dwelling older adults: the impact of subthreshold depression. *Psychol Med*. 2009; 39(10):1677-88.
36. Newsom JT, Schulz R. Social support as a mediator in the relation between functional status and quality of life in older adults. *Psychol Aging*. 1996; 11(1):34-44.
37. Kennedy GJ, Kelman HR, Thomas C. The emergence of depressive symptoms in late life: the importance of declining health and increasing disability. *J Community Health*. 1990; 15(2):93-104.
38. Lin IF, Wu HS. Does informal care attenuate the cycle of ADL/IADL disability and depressive symptoms in late life? *J Gerontol B Psychol Sci Soc Sci.* 2011; 66(5):585-94.
39. Jia LN, Zhuang HL, Wang XY, *et al*. Depression status of the elderly and its relationship with quality of life in community of Fuzhou city. *Chinese Journal of Geriatrics*. 2011; 30(11):970–973. (Chinese)
40. Bonanno GA, Westphal M, Mancini AD. Resilience in the face of loss and potential trauma. *Annu Rev Clin Psychol.* 2011; 7:511-35.
41. Assil S, Zeidan Z. Prevalence of depression and associated factors among elderly Sudanese: a household survey in Khartoum State. *East Mediterr Health J*. 2013; 19(5):435.
42. Wiltink J, Michal M, Wild PS, *et al.* Associations between depression and different measures of obesity (BMI, WC, WHtR, WHR). *BMC Psychiatry*. 2013;13:223.
43. Lopresti AL, Hood SD, Drummond PD. A review of lifestyle factors that contribute to

For peer review only

important pathways associated with major depression: diet, sleep, and exercise. *J Affect Disord*. 2013;148:12–27.

44. Lo A. Lifestyle factors on depression. In: Pachana N, editor. Encyclopedia of Geropsychology. Springer (2015)

Figure Legend

Figure 1 The importance sort and mechanism direction of physical disability with depressive symptoms by using explainable machine learning predictions

Figure 2 The importance sort and mechanism direction of number of comorbid chronic disease with depressive symptoms by using explainable machine learning predictions

For peer review only

Figure 1 The importance sort and mechanism direction of physical disability with depressive symptoms by using explainable machine learning predictions

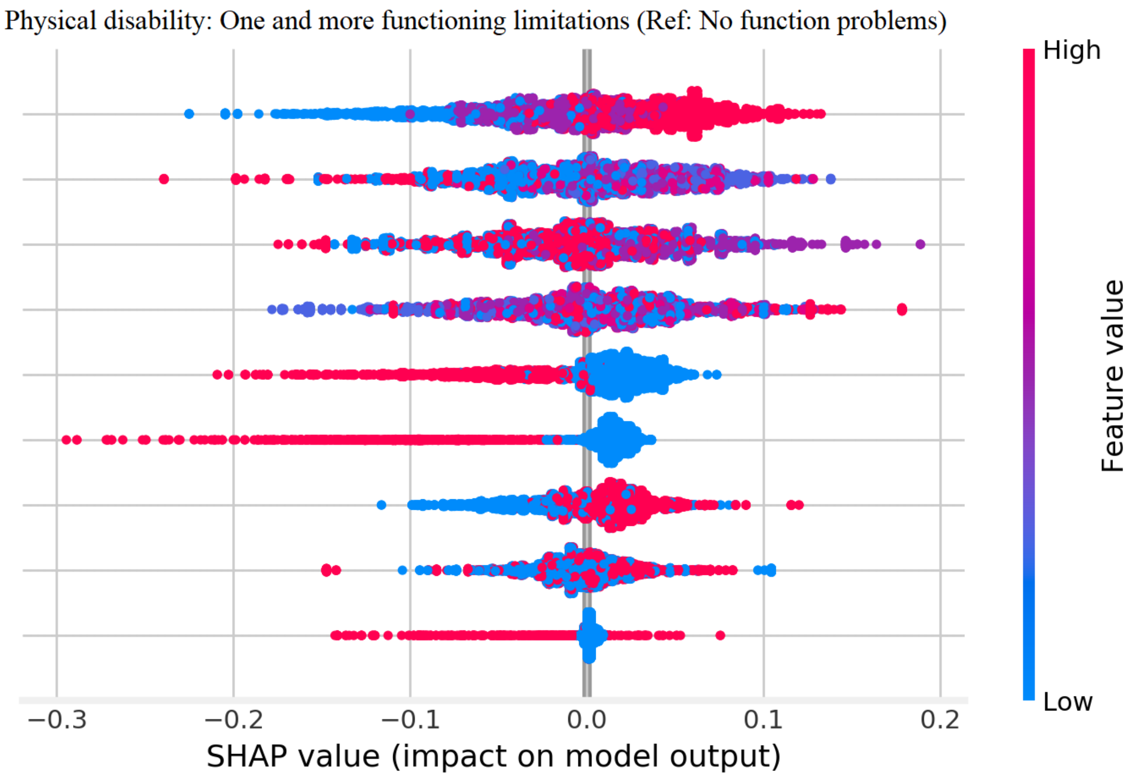

The importance of variables (Mean of SHAP value with 3 decimal places : Risk Factor (RF): mean of SHAP value > 0; Protective Factor (PF): mean of SHAP value < 0; No Effect (NE): mean of SHAP value = 0) by descending were education level (NE), age groups (RF), number of comorbid chronic disease (PF), wealth quantile (NE), marital status (PF), living arrangement (NE), residence (RF), gender (RF) and ethnicity (PF).

Figure 2 The importance sort and mechanism direction of number of comorbid chronic disease with depressive symptoms by using explainable machine learning predictions

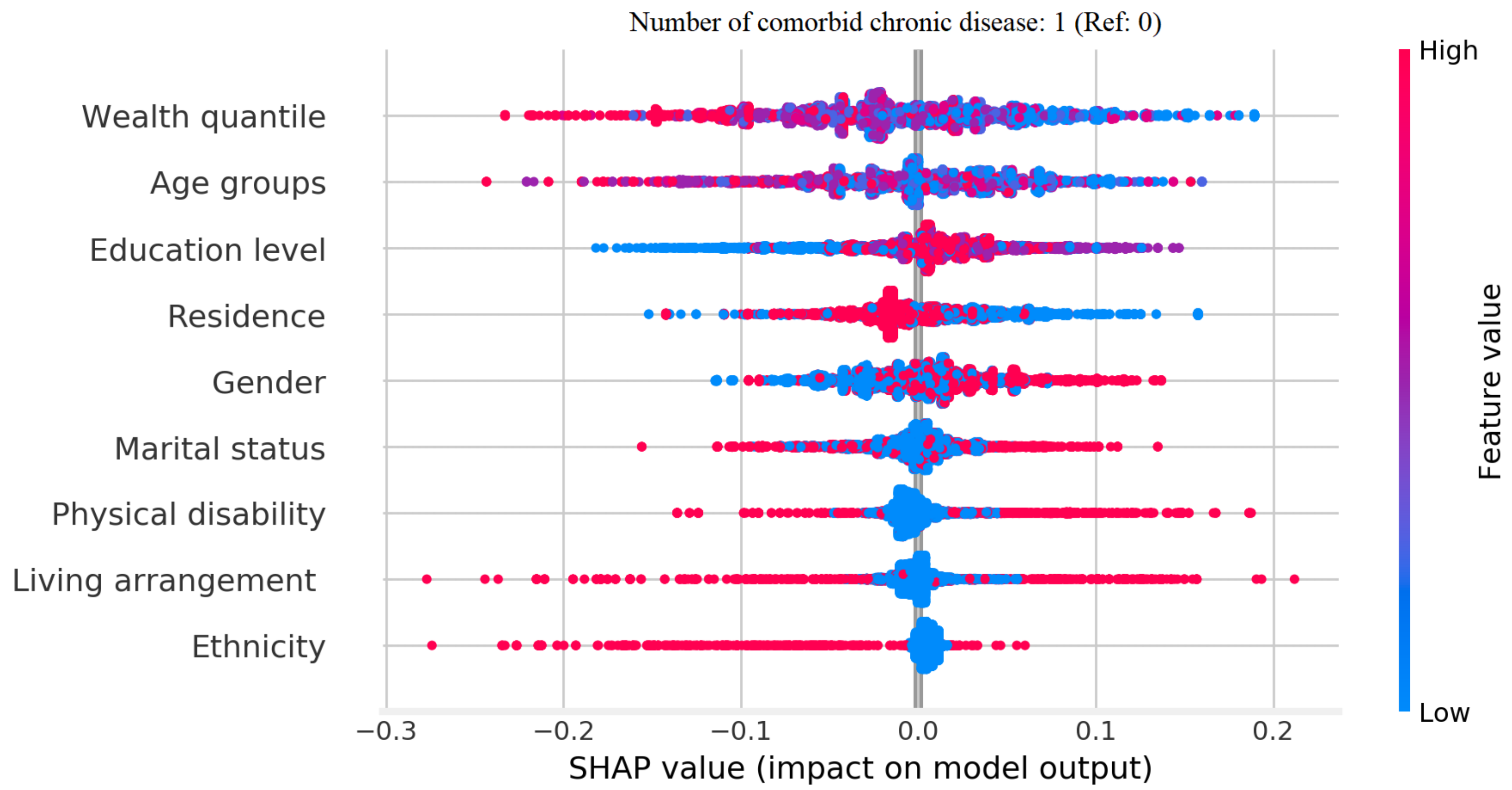

(a) Number of comorbid chronic disease: 1 (Ref: 0)

The importance of variables (Mean of SHAP value with 3 decimal places: Risk Factor (RF): mean of SHAP value > 0; Protective Factor (PF): mean of SHAP value < 0; No Effect (NE): mean of SHAP value = 0) by descending were wealth quantile (NE), age groups (RF), education level (RF), residence (RF), gender (RF), marital status (PF), physical disability (RF), living arrangement (NE) and ethnicity (PR).

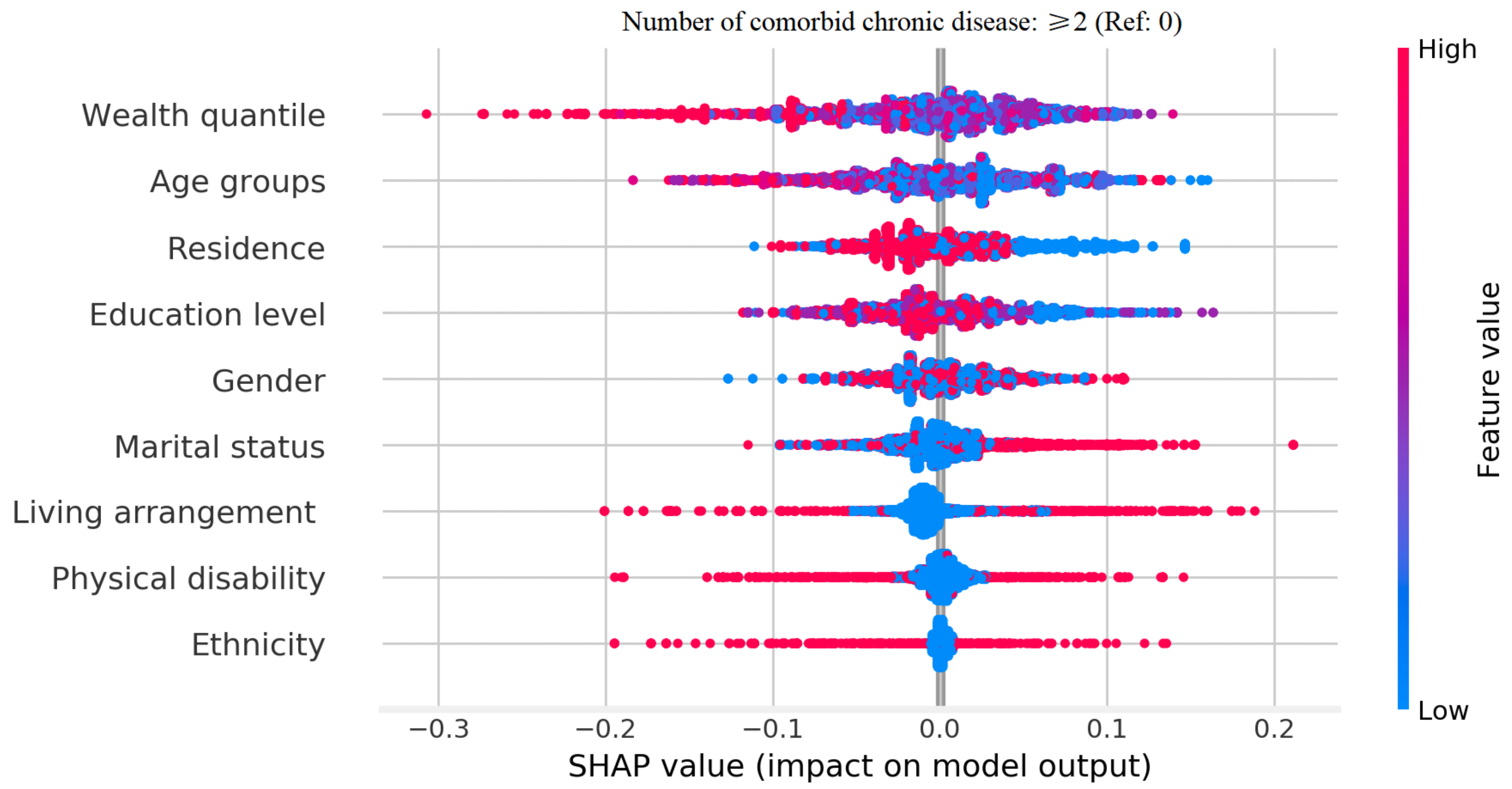

(b) Number of comorbid chronic disease: ⩾2 (Ref: 0)

The importance of variables (Mean of SHAP value with 3 decimal places: Risk Factor (RF): mean of SHAP value > 0; Protective Factor (PF): mean of SHAP value < 0; No Effect (NE): mean of SHAP value = 0) by descending were wealth quantile (PF), age groups (RF), residence (RF), education level (NE), gender (RF), marital status (NF), living arrangement (NE), physical disability (NF) and ethnicity (PF).

STROBE Statement—Checklist of items that should be included in reports of ***cross-sectional studies***

	Item No	Recommendation	Page No
**Title and abstract**	1	(*a*) Indicate the study's design with a commonly used term in the title or the abstract	1
		(*b*) Provide in the abstract an informative and balanced summary of what was done and what was found	2-3
**Introduction**			
Background/rationale	2	Explain the scientific background and rationale for the investigation being reported	4
Objectives	3	State specific objectives, including any prespecified hypotheses	4
**Methods**			
Study design	4	Present key elements of study design early in the paper	5
Setting	5	Describe the setting, locations, and relevant dates, including periods of recruitment, exposure, follow-up, and data collection	5
Participants	6	(*a*) Give the eligibility criteria, and the sources and methods of selection of participants	5
Variables	7	Clearly define all outcomes, exposures, predictors, potential confounders, and effect modifiers. Give diagnostic criteria, if applicable	5-6
Data sources/ measurement	8*	For each variable of interest, give sources of data and details of methods of assessment (measurement). Describe comparability of assessment methods if there is more than one group	5-6
Bias	9	Describe any efforts to address potential sources of bias	
Study size	10	Explain how the study size was arrived at	5
Quantitative variables	11	Explain how quantitative variables were handled in the analyses. If applicable, describe which groupings were chosen and why	
Statistical methods	12	(*a*) Describe all statistical methods, including those used to control for confounding	6-8
		(*b*) Describe any methods used to examine subgroups and interactions	6-8
		(*c*) Explain how missing data were addressed	6-8
		(*d*) If applicable, describe analytical methods taking account of sampling strategy	6-8
		(*e*) Describe any sensitivity analyses	6-8
**Results**			
Participants	13*	(a) Report numbers of individuals at each stage of study—eg numbers potentially eligible, examined for eligibility, confirmed eligible, included in the study, completing follow-up, and analysed	7-8
		(b) Give reasons for non-participation at each stage	7-8
		(c) Consider use of a flow diagram	7-8
Descriptive data	14*	(a) Give characteristics of study participants (eg demographic, clinical, social) and information on exposures and potential confounders	7-8
		(b) Indicate number of participants with missing data for each variable of interest	7-8
Outcome data	15*	Report numbers of outcome events or summary measures	7-8
Main results	16	(*a*) Give unadjusted estimates and, if applicable, confounder-adjusted estimates and their precision (eg, 95% confidence interval). Make clear which confounders were adjusted for and why they were included	7-8

For peer review only - http://bmjopen.bmj.com/site/about/guidelines.xhtml

		(*b*) Report category boundaries when continuous variables were categorized	7-8
		(*c*) If relevant, consider translating estimates of relative risk into absolute risk for a meaningful time period	7-8
Other analyses	17	Report other analyses done—eg analyses of subgroups and interactions, and sensitivity analyses	7-8
**Discussion**			
Key results	18	Summarise key results with reference to study objectives	8-10
Limitations	19	Discuss limitations of the study, taking into account sources of potential bias or imprecision. Discuss both direction and magnitude of any potential bias	10
Interpretation	20	Give a cautious overall interpretation of results considering objectives, limitations, multiplicity of analyses, results from similar studies, and other relevant evidence	8-11
Generalisability	21	Discuss the generalisability (external validity) of the study results	11-12
**Other information**			
Funding	22	Give the source of funding and the role of the funders for the present study and, if applicable, for the original study on which the present article is based	

*Give information separately for exposed and unexposed groups.

Note: An Explanation and Elaboration article discusses each checklist item and gives methodological background and published examples of transparent reporting. The STROBE checklist is best used in conjunction with this article (freely available on the Web sites of PLoS Medicine at http://www.plosmedicine.org/, Annals of Internal Medicine at http://www.annals.org/, and Epidemiology at http://www.epidem.com/). Information on the STROBE Initiative is available at www.strobe-statement.org.

For peer review only

For peer review only - http://bmjopen.bmj.com/site/about/guidelines.xhtml

BMJ Open

Estimating the causal effects of physical disability and number of comorbid chronic diseases on depressive symptom risk in an elderly Chinese population: A machine learning analysis of cross-sectional baseline data from the China Longitudinal Ageing Social Survey

Journal:	*BMJ Open*
Manuscript ID	bmjopen-2022-069298.R1
Article Type:	Original research
Date Submitted by the Author:	28-Mar-2023
Complete List of Authors:	Wang, Zhenjie; Peking University<br>Yang, Hanmo; Harvard University<br>Sun, Chenxi; Peking University; Peking University<br>Hong, Shenda; Peking University; Health Science Center of Peking University
**Primary Subject Heading**:	Health informatics
Secondary Subject Heading:	Health informatics
Keywords:	Health informatics < BIOTECHNOLOGY & BIOINFORMATICS, MENTAL HEALTH, Depression & mood disorders < PSYCHIATRY

SCHOLARONE™
Manuscripts

I, the Submitting Author has the right to grant and does grant on behalf of all authors of the Work (as defined in the below author licence), an exclusive licence and/or a non-exclusive licence for contributions from authors who are: i) UK Crown employees; ii) where BMJ has agreed a CC-BY licence shall apply, and/or iii) in accordance with the terms applicable for US Federal Government officers or employees acting as part of their official duties; on a worldwide, perpetual, irrevocable, royalty-free basis to BMJ Publishing Group Ltd ("BMJ") its licensees and where the relevant Journal is co-owned by BMJ to the co-owners of the Journal, to publish the Work in this journal and any other BMJ products and to exploit all rights, as set out in our licence.

The Submitting Author accepts and understands that any supply made under these terms is made by BMJ to the Submitting Author unless you are acting as an employee on behalf of your employer or a postgraduate student of an affiliated institution which is paying any applicable article publishing charge ("APC") for Open Access articles. Where the Submitting Author wishes to make the Work available on an Open Access basis (and intends to pay the relevant APC), the terms of reuse of such Open Access shall be governed by a Creative Commons licence – details of these licences and which Creative Commons licence will apply to this Work are set out in our licence referred to above.

Other than as permitted in any relevant BMJ Author's Self Archiving Policies, I confirm this Work has not been accepted for publication elsewhere, is not being considered for publication elsewhere and does not duplicate material already published. I confirm all authors consent to publication of this Work and authorise the granting of this licence.

For peer review only

Estimating the causal effects of physical disability and number of comorbid chronic diseases on depressive symptom risk in an elderly Chinese population: A machine learning analysis of cross-sectional baseline data from the China Longitudinal Ageing Social Survey

Zhenjie Wang,[1*] Hanmo Yang,[4] Chenxi Sun,[5,6] Shenda Hong[2,3*]

[1] Institute of Population Research, Peking University, Beijing 100871, People's Republic of China

[2] National Institute of Health Data Science, Peking University, Beijing 100191, People's Republic of China

[3] Institute of Medical Technology, Health Science Center of Peking University, Beijing 100191, People's Republic of China

[4] T. H. Chan School of Public Health, Harvard University, Boston 02115, United States of America

[5] School of Electronics Engineering and Computer Science, Peking University, Beijing, People's Republic of China

[6] Key Laboratory of Machine Perception (Ministry of Education), Peking University, Beijing, People's Republic of China

[*] **Correspondence:** Zhenjie Wang, Institute of Population Research, Peking University, No. 5 Yiheyuan Road, Haidian District, Beijing 100871, People's Republic of China; E-mail: zhenjie.wang@pku.edu.cn. Shenda Hong, National Institute of Health Data Science, Peking University, Beijing 100191, People's Republic of China；E-mail: hongshenda@pku.edu.cn

For peer review only

ABSTRACT

Objective This study aimed to explore the causal effects of physical disability and number of comorbid chronic diseases on depressive symptoms in an elderly Chinese population.

Design, setting and analysis Data were obtained from the China Longitudinal Ageing Social Survey (CLASS), a stratified, multistage, probabilistic sampling survey conducted in 2014 that covers 28 of 31 provincial areas in China. The causal effects of physical disability and number of comorbid chronic diseases on depressive symptoms were analyzed using the conditional average treatment effect (CATE) method of machine learning.

Outcome The outcome for this study was an assessment of the causal effects of physical disability and number of comorbid chronic diseases on depressive symptoms.

Participants A total of 7496 subjects who were 60 years of age or older and who answered the questions on depressive symptoms and other independent variables of interest in a survey conducted in 2014 were included in this study.

Results Physical disability and number of comorbid chronic diseases had causal effects on depressive symptoms (DS). Among the subjects who had one and more functional limitations, the possibility of DS increased by 22%. The subjects who had one chronic disease and two or more chronic diseases, the possibility of DS increased by 13%, and 20%, respectively.

Conclusion: The present study provides evidence that the presence of one or more functional limitations affects the occurrence of depressive symptoms among elderly people. This information is helpful in planning possible preventive measures and in improving knowledge of how to treat depressive symptoms. The findings of our study are of value in developing programs that are designed to identify elderly individuals who have physical disabilities or

For peer review only

comorbid chronic diseases and to provide early intervention.

Keywords: Health informatics, mental health, causal effects, older population, Chinese

Strengths and limitations of this study

- The current study used a large, representative population-based sample covering 28 of 31 provincial areas in China.
- The study estimated causal effects using the conditional average treatment effect (CATE) method.
- The findings of this cross-sectional study established a cause-and-effect relationship based on the use of a machine learning algorithm. However, the cross-sectional design of the baseline of the CLASS should be taken into account in further research.
- In the current study, we were unable to evaluate the effects of lifestyle factors since information on these factors was not collected in the CLASS. This must also be considered in further research.

For peer review only

INTRODUCTION

Currently, depressive symptoms (DS) are one of the most prevalent mental disorders worldwide.[1] The characteristics of DS include symptoms of sadness, depressed mood, and loss of interest.[1] The presence of DS is usually associated with low quality of life, cancer, chronic diseases, suicide, and other conditions.[2-5] DS can also place a heavy burden on families, communities and health services worldwide.[6, 7] The prevalence of DS varies widely and ranges from 1% to 16% among middle-aged and elderly populations.[8-10] In China, the prevalence of DS among elderly people has increased rapidly from 3.9% to 17% in the past two decades.[11-13]

Many studies have provided evidence that specific demographic variables are associated with DS risk,[14] although the associations are inconsistent.[10, 13] An inverse or U-shaped association between age and DS has been found in many studies.[15-17] Demographic status and level of education were also found to be associated with DS risk.[18-20] However, some studies reported no association of DS with demographic status or level of education.[14,17,21]

Very few studies have used machine learning to obtain evidence regarding the causal effects of physical disability or the number of comorbid chronic diseases on DS risk in the elderly Chinese population. In this study, we used machine learning to explore the conditional average treatment effect (CATE) of physical disability and number of comorbid chronic diseases on depressive symptoms using data from the Chinese Longitudinal Ageing Social Survey (CLASS).

METHODS AND MATERIALS

For peer review only

Data source

We drew the sample used in the current study from the Chinese Longitudinal Ageing Social Survey (CLASS), which was conducted in 2014.[22] The CLASS was collected by the National Survey Research Center, Renmin University. The CLASS used a stratified, multistage, probabilistic sampling method to select a nationally representative sample covering 28 of 31 provincial areas in China. A total of 11,511 older adults were surveyed. In the present study, the sample comprised 7496 subjects 60 years of age or older who answered the questions on depressive symptoms and other independent variables of interest collected in 2014. All the participants were interviewed face-to-face by trained interviewers.

Measurement of Variables

Depressive symptoms

Depressive symptoms were assessed using the 9-item Center for Epidemiological Studies Depression Scale (CES-D). This scale includes three items designed to assess positive feelings and two items each designed to assess negative emotions, somatic symptoms, and sense of marginalization. The 9-item CES-D is reliable and valid for detecting nonpsychotic mental disorders among older Chinese adults.[23] For each item, a score of 0 (rarely or none of the time), 1 (some of the time), or 2 (most of the time) was assigned; thus, the total score ranged from 0 to 18. Since the coding of the positive effect items was reversed, a higher score indicates a higher level of depressive symptoms. In the current study, on the 9-item scale, the total possible score was 18 (9 items multiplied by 2, the highest response). A standardized cutoff score of 4.8 for the 9-item form of the CES-D was established using Kohout's

For peer review only

formula.[24] In this study, the internal Cronbach's alpha for the nine items was 0.75.

Physical disabilities

Activities of daily living (ADLs) were assessed using the Barthel Index. For each individual, the self-reported number of comorbid chronic diseases, including health problems such as hypertension, diabetes, heart disease, renal disease, liver disease, stroke, tuberculosis, arthritis, and respiratory diseases, was categorized as "0", "1" or "≥2".

Confounding variables

The recorded demographic characteristics included gender (male, female), age, marital status (married, widowed/divorced/unmarried), ethnicity (Han, others), residence (rural, urban), educational level (junior high school and above, primary school, never attended school), and living arrangements (lives alone, lives with others). Income was categorized into five levels using quintiles of household income (yuan) (Q1: ≤ 3000; Q2: >3000 and ≤ 10000; Q3: >10000 and ≤ 24000; Q4: >24000 and ≤ 36000; Q5: >36000).

Estimation of causal effects

Causal analysis aims to determine the causal relationships between variables (treatments) and outcomes. Causal relationships are essentially different from correlations in that causal relationships mainly solve the problem of "why". In this work, we used Causal ML,an open-source Python software provided by Uber, to perform causal analysis using machine learning algorithms based on recent research.[25] Causal ML provides a standard interface that allows

For peer review only

users to estimate the average treatment effect (ATE) based on experimental or observational data.

To estimate the causal relationship between the variable of interest (known as treatment indicator E) and the outcome Y, we performed a two-step analysis: (1) we built a machine learning model and (2) we estimated the ATE based on causal ML. First, we built a random forest classifier (RFC) and used it to predict DS using all the variables except the treatment indicator (denoted X).[26] The RFC contains 100 decision trees and uses the Gini purity index to judge the segmentation criteria of a tree node. The maximum depth of a single tree was set to unlimited so that each tree would be segmented until each leaf node of the tree model contained only one sample. The RFC output was 1 if the individual had DS and 0 otherwise. The RFC model was built using a scikit-learn machine learning framework.[27]

We then set the above trained RFC as the base learner for causal ML and estimated the treatment effect based on the algorithm described herein.[28] The algorithm, which uses a single base learner from the machine learning model and has the treatment indicator as a feature, operates in two stages. In the first stage, the algorithm estimates the average outcomes after specific treatments using the trained RFC model, where the outcome is calculated as $\mu(x) = E(Y|X = x, E = e)$. In the second stage, the ATE was calculated as $\tau(x) = \mu(x, E = 1) - \mu(x, E = 0)$. We also used SHAPley Additive exPlanations (SHAP), a game theory approach, to explain the output of the causal analysis results modeled by the machine learning models.[29] We analyzed the data using Python 3.7.14 based on the anaconda3 development platform, causalml version 0.10.0, sklearn version 0.23.2, pandas version 1.0.1, and numpy version 1.21.0.

For peer review only

Survey conduct and consent

The interviewer-administered questionnaire was prepared in the local language (Chinese). Consent to participation in the study was received from each participant prior to data collection, and the data collectors were trained to provide any requested information or clarification at any time during the interview. Only participants who wished to continue participating in the study after providing informed consent were included in the study.

Patient and public involvement

No patient or public involvement.

RESULTS

Descriptive statistics

The characteristics of subjects with and without depressive symptoms (DS) are summarized in Table 1. The overall average Center for Epidemiological Studies Depression Scale (CES-D) score was 4.56 (standard deviation: 3.56). The prevalence of DS in the Chinese population over 60 years of age was 43%. In the current study, male subjects, urban residents, people living with others, and people of Han ethnicity accounted for the majority of elderly Chinese individuals with DS.

Table 1 Subjects' characteristics by their depressive symptom status according to the 9-item Center for Epidemiological Studies Depression Scale (CES-D) among the elderly population in China

Characteristics	Lower than depressive	Higher than depressive	*P*

For peer review only

	symptom cut-off point*	symptom cut-off point*	
Age (years)			
60-64	1603 (37.2)	1068 (33.5)	< 0.001
65-69	988 (22.9)	704 (22.1)	
70-74	766 (17.8)	547 (17.2)	
75-79	530 (12.3)	471 (14.8)	
80+	422 (9.8)	397 (12.5)	
Residence			
Rural	1207 (28.0)	1315 (41.3)	< 0.001
Urban	3102 (72.0)	1872 (58.8)	
Gender			
Male	2427 (56.3)	1627 (51.1)	< 0.001
Female	1882 (43.7)	1563 (48.9)	
Marital status			
Married	3316 (77.0)	2038 (63.9)	< 0.001
Widowed/divorced/unmarried	993 (23.0)	1149 (36.1)	
Educational level			
Never attended school	651 (15.1)	816 (25.6)	< 0.001
Primary school	1409 (32.7)	1266 (39.7)	
Junior high school and above	2249 (52.2)	1105 (34.7)	
Ethnicity			
Han	4074 (94.5)	2990 (93.8)	0.19
Other	235 (5.5)	197 (6.2)	
Living arrangement			
Live with others	3925 (91.1)	2666 (83.7)	< 0.001
Live alone	384 (8.9)	521 (16.3)	
Physical disability			
No functional limitations	3988 (92.6)	2531 (79.4)	< 0.001
One or more functional limitations	321 (7.4)	656 (20.6)	
Wealth quantile			
Q1 (lowest)	691 (16.0)	925 (29.0)	< 0.001
Q2	694 (16.1)	717 (22.5)	
Q3	1066 (24.7)	705 (22.1)	
Q4	1002 (23.3)	520 (16.3)	
Q5 (highest)	856 (19.9)	320 (10.1)	
Number of comorbid chronic diseases			
0	1463 (34.0)	551 (17.3)	< 0.001
1	1307 (30.3)	917 (28.8)	
≥2	1539 (35.7)	1719 (53.9)	

* For the current study, on a 9-item scale, the total possible score was 18 (9 items multiplied by 2, the highest response). The standardized cut-off point was 4.8 by using Kohout's formula.

For peer review only

Estimated causal effects

The estimated causal effects of physical disability and chronic diseases on depressive symptoms are presented in Table 2. Physical disability and the number of comorbid chronic diseases had causal effects on DS. Among the subjects who had one or more functional limitations, those who had one chronic disease, and those who had two or more chronic diseases, the risk probability of DS increased by 22%, 13%, and 20%, respectively.

Table 2 Estimated causal effects of physical disability and chronic disease on depressive symptoms

	Reference		Causal effect (95% CI)
Physical disability *	No functional problems	One or more functional limitations	0.22 (0.19, 0.24)
Number of comorbid chronic diseases†	0	1	0.13 (0.10, 0.15)
		≥2	0.20 (0.18, 0.22)

* taking into consideration the causal effects of age, gender, residence, marital status, educational level, ethnicity, wealth quantile, and number of comorbid chronic diseases.
† taking into consideration the causal effects of age, gender, residence, marital status, educational level, ethnicity, wealth quantile, and physical disability.

The effects of the mechanism direction of physical disability and number of comorbid chronic diseases on DS are presented in Figures 1-2 in descending order of importance In descending order of importance among subjects who had one or more functional limitations, the variables are as follows: educational level, age, number of comorbid chronic diseases, wealth quantile, marital status, living arrangement, residence, gender and ethnicity. In descending order of importance among subjects who had only one chronic disease, the variables are as follows: wealth quantile, age, educational level, residence, gender, marital status, physical disability, living arrangement and ethnicity. In descending order of importance among subjects who had one or more functional limitations, the variables are as follows: wealth quantile, age, residence, educational level, gender, marital status, living arrangement, physical disability and ethnicity. Age, residence, gender, and physical disability

For peer review only

contributed to DS risk among subjects who had one or more chronic diseases.

DISCUSSION

Overall, physical disability and the number of comorbid chronic diseases present had causal effects on depressive symptom (DS) risk. Subjects who had one or more functional limitations had a 22% increased risk of DS. Subjects who had one chronic disease and those who had two or more chronic diseases showed increases of 13% and 20%, respectively, in their DS risk.

In the current study, we found causal effects of physical disability and comorbid chronic diseases on DS. A previous study suggested that some health variables, such as poor self-rated health and disability, are associated with DS, indicating that health status tends to have similar effects on mental health regardless of social context.[30] Physical disability is considered an inability or a diminished capacity to perform basic self-care activities. Physical disability lessens an individual's ability to interact with the physical and social environment, hence increasing the risk of DS.[31] Increased disability in activities of daily living (ADLs) and instrumental activities of daily living (IADLs) have been proven to be connected with depressive symptoms.[32,33] Failure to perform ADLs has been positively associated with depressive symptoms in elderly people, and physical disability accounts for incidences of depressive symptoms in elderly individuals.[34,35] Furthermore, the finding that physical disability leads to an increase in depressive symptoms in geriatric individuals over time is supported by longitudinal studies.[36] Given the influence of physical disability and depressive symptoms on the well-being of elderly individuals, it is urgent to find ways to address these issues. The results presented here demonstrate that impairment of ADLs has a significant effect on depressive symptoms in elderly people, consistent with the findings of previous

For peer review only

studies that showed a relationship between limitations on activity and psychosocial problems.[31,32]

The number of chronic diseases present in an individual was found to be associated with DS risk, consistent with previous studies. These associations could be explained by several factors, including unhealthy lifestyle, shared genetic influences, and inflammation related to disease.[37] Previous studies have suggested that experiencing pain and physical disability could affect mood in elderly individuals.[20] Another explanation is that subjects with depressive symptoms might be more sedentary than those without depressive symptoms; such a lifestyle could cause increased bone resorption and decreased bone formation.[38,39]

Strengths and limitations

Our study has many strengths, including a large sample size, a population-based design, and adjustment for a wide range of socioeconomic characteristics. Another noticeable strength is that the measurement of all physical illnesses took place prior to the CES-D measurement, minimizing the risk of reverse causation. However, our study also has several limitations that should be taken into account by future researchers. First, although we used machine learning to estimate the causal effects of the factors on DS, these findings should be interpreted cautiously in view of the cross-sectional nature of the data used in the present analysis. We will be able to extend the current study to determine causality when longitudinal data are available. Although the current data were collected in 2014, they are available on the web. Because all the information was collected by trained interviewers, the quality of the survey was reliable. This point should also be considered with caution by future researchers. It is

For peer review only

important to note that the CLASS does not provide information on lifestyle factors (i.e., weight, height, smoking, alcohol consumption, and other factors), some of which have been suggested as risk factors for depression.[40-42]

CONCLUSION

In conclusion, in this large population-based study in an elderly Chinese population, physical disability and the number of comorbid chronic diseases were found to increase DS risk. The present study provides evidence of the effect of one or more functional limitations on the occurrence of depressive symptoms among elderly people. This is helpful in planning preventive measures and in improving knowledge of how to treat depressive symptoms. The findings made in our study are valuable for the development of prevention programs designed to identify elderly individuals who have physical disabilities or a number of comorbid chronic diseases and provide early intervention.

Acknowledgments We would like to thank the Institute of Gerontology and National Survey Research Center at Renmin University of China for providing the CLASS data.

Contributors Conceptualization: Z. W.; Methodology: S. H.; Software: Z. W. and S. H.; Writing: Z. W.; Writing—review: Z. W., S. H., H. Y. and C. S.; Writing—editing: H. Y. and C. S.

Funding This work was supported by the National Social Science Fund of China (No. 20BRK020), the National Natural Science Foundation of China (No. 62102008) and the

For peer review only

Economic & Social Research Council (ESRC) (No. ES/P011055/1). The funding agencies did not participate in the research or review any details of this study, and the authors are independent of the funders.

Competing interests None declared.

Patient consent for publication Not needed.

Ethics approval All procedures performed in this study involving human participants were conducted in accordance with the ethical standards of the institutional and/or national research committee and with the 1964 Helsinki Declaration and its later amendments or comparable ethical standards. The surveys were conducted within the legal framework governed by statistical law in China. This survey was approved by Renmin University of China. Our group received permission for use of the CLASS data from the National Survey Research Center at Renmin University of China. This article does not involve any animal studies.

Provenance and peer review Not commissioned; externally peer reviewed.

Data availability statement The datasets generated and/or analyzed during the current study are available at the China Longitudinal Ageing Social Survey (CLASS) repository, http://class.ruc.edu.cn/index.php?r=index/index&hl=en.

REFERENCES

1. Whiteford HA, Ferrari AJ, Degenhardt L, *et al*. Global Burden of Mental, Neurological, and Substance Use Disorders: An Analysis from the Global Burden of Disease Study 2010. *PLoS One*. 2015;10:e0116820.

2. Dalton SO, Laursen TM, Ross L, *et al*. Risk for hospitalization with depression after a

For peer review only

cancer diagnosis: a nationwide, population-based study of cancer patients in Denmark from 1973 to 2003. *J Clin Oncol.* 2009; 27:1440–1445.

3. Hsu YT, Liao CC, Chang SN, Y *et al*. Increased Risk of Depression in Patients with Parkinson Disease: A Nationwide Cohort Study. *Am J Geriatr Psychiatry.* 2015; 23:934–940.
4. Ayerbe L, Ayis S, Wolfe CD, *et al*. Natural history, Predictors and outcomes of depression after stroke: systematic review and meta-analysis. *Br J Psychiatry.* 2013; 202:14-21.
5. Cuijpers P, Vogelzangs N, Twisk J, *et al*. Comprehensive meta-analysis of excess mortality in depression in the general community versus patients with specific illnesses. *Am J Psychiatry.* 2014; 171: 453–462.
6. World Health Organization. Depression–a global public health concern. 2012, http://hesp-news.org/2012/10/05/depression-a-global public-health-concern/ (accessed: 2018-11-29).
7. Walker ER, McGee RE, Druss BG. Mortality in mental disorders and global disease burden implications: a systematic review and meta-analysis. *JAMA Psychiatry.* 2015; 72:334–341.
8. Wilhelm K, Mitchell P, Slade T, *et al*. Prevalence and correlates of DSM-IV major depression in an Australian national survey. *J Affect Disord.* 2003; 75:155–162.
9. Waraich P, Goldner E M, Somers J M, *et al*. Prevalence and incidence studies of mood disorders: a systematic review of the literature. *Can J Psychiatry*. 2004; 49:124–138.
10. Djernes JK. Prevalence and predictors of depression in populations of elderly: a review. *Acta Psychiatr Scand.* 2006;113:372–387.

For peer review only

11. Chen R, Copeland J R, Wei L. A meta-analysis of epidemiological studies in depression of older people in the People's Republic of China. *Int J Geriatr Psychiatry.* 1999; 14:821–830.

12. Li D, Zhang DJ, Shao JJ, *et al*. A meta-analysis of the prevalence of depressive symptoms in Chinese older adults. *Arch Gerontol Geriatr*. 2014; 58:1–9.

13. Zhang L, Xu Y, Nie H, *et al*. The prevalence of depressive symptoms among the older in China: a meta-analysis. *Int J Geriatr Psychiatry.* 2012; 27:900–906.

14. Gao S, Jin Y, Unverzagt FW, *et al*. Correlates of depressive symptoms in rural elderly Chinese. *Int J Geriatr Psychiatry.* 2009; 24:1358–1366.

15. Kessler RC, Birnbaum HG, Shahly V, *et al*. Age differences in the prevalence and co-morbidity of DSM-IV major depressive episodes: results from the WHO World Mental Health Survey Initiative. *Depress Anxiety.* 2010; 27:351–364.

16. Pan A, Franco OH, Wang YF, *et al*. Prevalence and geographic disparity of depressive symptoms among middle-aged and elderly in China. *J Affect Disord.* 2008;105:167–175.

17. Yunming L, Changsheng C, Haibo T, *et al*. Prevalence and risk factors for depression in older people in Xi'an China: a community-based study. *Int J Geriatr Psychiatry.* 2012; 27(1):31-9.

18. Lorant V, Croux C, Weich S, *et al*. Depression and socio-economic risk factors: 7-year longitudinal population study. *Br J Psychiatry*. 2007; 190:293–298.

19. Van d V, Bracke P, Levecque K. Gender differences in depression in 23 European countries. Cross-national variation in the gender gap in depression. *Soc Sci Med*. 2010; 71:305–313.

For peer review only

20. Zhou X, Bi B, Zheng L, *et al*. The prevalence and risk factors for depression symptoms in a rural Chinese sample population. *PLoS One.* 2014; 9:e99692.

21. van der Wurff F B, Beekman A T, *et al*. Prevalence and risk-factors for depression in elderly Turkish and Moroccan migrants in the Netherlands. *J Affect Disord.* 2004; 83:33–41.

22. The Chinese Longitudinal Ageing Social study. Available: http://class.ruc.edu.cn/English/Home.htm

23. Silverstein M, Cong Z, Li S. Intergenerational transfers and living arrangements of older people in rural China: consequences for psychological wellbeing. J. Gerontol. 2006; 61:S256–S266.

24. Kohout FJ, Berkman LF, Evans DA, Cornoni-Huntley J. Two shorter forms of the CES-D depression symptoms index. *J Aging Health.* 1993;5(2):179–193.

25. Chen H, Harinen T, Lee JY, *et al*. Causalml: python package for causal machine learning. *arXiv.* (2020).

26. Breiman L ."Random Forests." *Machine Learning*. 2001; 45(1), 5-32.

27. Pedregosa F, Varoquaux G, Gramfort A, *et al*. Scikit-learn: machine learning in python. Scikit-learn: Machine learning in Python. *Journal of machine Learning research*. 2012;12: 2825-2830.

28. Künzel Sren R, Sekhon JS, Bickel PJ, Yu B. Meta-learners for estimating heterogeneous treatment effects using machine learning. *Proc Natl Acad Sci U S A.* 2017; 116: 4156-4165.

29. Lundberg S, Lee SI. A Unified Approach to Interpreting Model Predictions. In

For peer review only - http://bmjopen.bmj.com/site/about/guidelines.xhtml

Proceedings of the 31st international conference on neural information processing system. 2017; 4768-4777.

30. Kino S, Jang SN, Kawachi I. Healthy but unhappy? Cross-national comparative analysis of depressive symptoms in Japanese vs. Korean elders. *Arch Gerontol Geriatr.* 2021; 95:104426.

31. Bruce ML. Depression and disability in late life: directions for future research. *Am J Geriatr Psychiatry*. 2001;.9(2):102-12.

32. Bowen ME, Ruch A. Depressive Symptoms and Disability Risk Among Older White and Latino Adults by Nativity Status. *J Aging Health*. 2015; 27(7):1286-305.

33. Hybels CF, Pieper CF, Blazer DG. The complex relationship between depressive symptoms and functional limitations in community-dwelling older adults: the impact of subthreshold depression. *Psychol Med*. 2009; 39(10):1677-88.

34. Newsom JT, Schulz R. Social support as a mediator in the relation between functional status and quality of life in older adults. *Psychol Aging*. 1996; 11(1):34-44.

35. Kennedy GJ, Kelman HR, Thomas C. The emergence of depressive symptoms in late life: the importance of declining health and increasing disability. *J Community Health*. 1990; 15(2):93-104.

36. Lin IF, Wu HS. Does informal care attenuate the cycle of ADL/IADL disability and depressive symptoms in late life? *J Gerontol B Psychol Sci Soc Sci.* 2011; 66(5):585-94.

37. Jia LN, Zhuang HL, Wang XY, *et al*. Depression status of the elderly and its relationship with quality of life in community of Fuzhou city. *Chinese Journal of Geriatrics*. 2011; 30(11):970–973. (Chinese)

For peer review only

38. Bonanno GA, Westphal M, Mancini AD. Resilience in the face of loss and potential trauma. *Annu Rev Clin Psychol.* 2011; 7:511-35.

39. Assil S, Zeidan Z. Prevalence of depression and associated factors among elderly Sudanese: a household survey in Khartoum State. *East Mediterr Health J*. 2013; 19(5):435.

40. .Wiltink J, Michal M, Wild PS, et al. Associations between depression and different measures of obesity (BMI, WC, WHtR, WHR). *BMC Psychiatry*. 2013;13:223.

41. Lopresti AL, Hood SD, Drummond PD. A review of lifestyle factors that contribute to important pathways associated with major depression: diet, sleep, and exercise. *J Affect Disord*. 2013;148:12–27.43.

42. Lo A. Lifestyle factors on depression. In: Pachana N, editor. Encyclopedia of Geropsychology. Springer (2015)

Figure Legend

Figure 1 The importance sort and mechanism direction of physical disability with depressive symptoms by using explainable machine learning predictions

Figure 2 The importance sort and mechanism direction of number of comorbid chronic disease with depressive symptoms by using explainable machine learning predictions

For peer review only

Figure 1 The importance sort and mechanism direction of physical disability with depressive symptoms by using explainable machine learning predictions

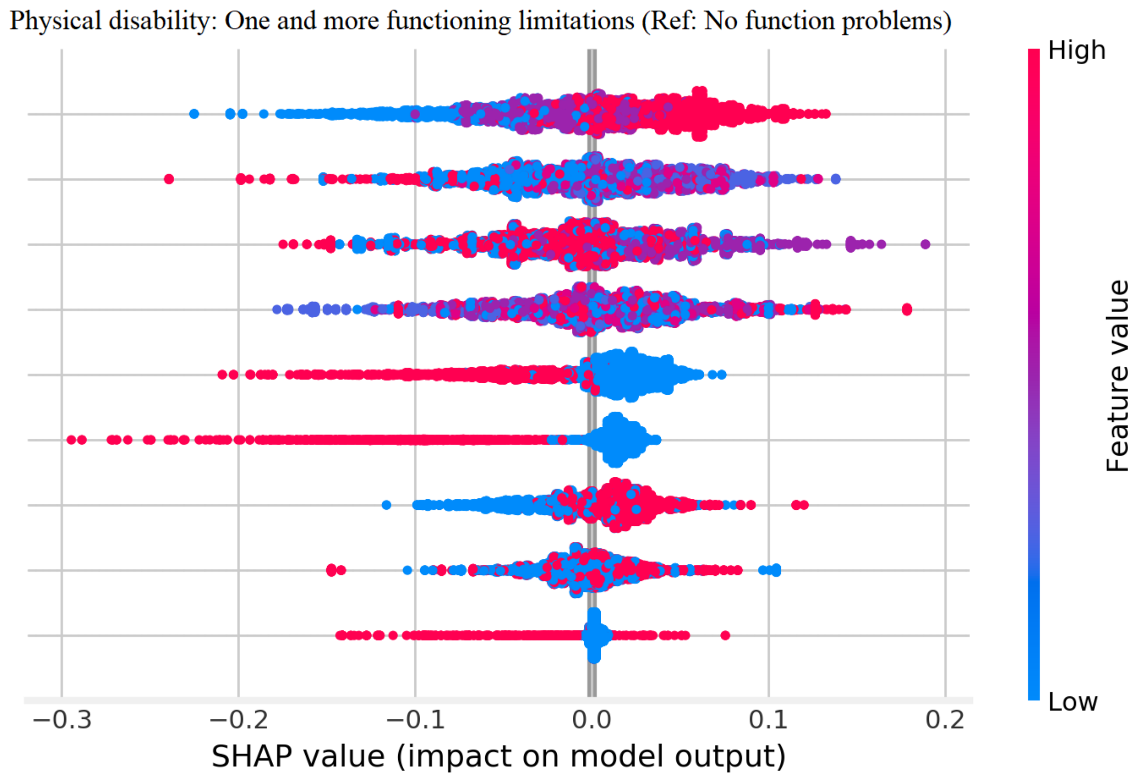

The importance of variables (Mean of SHAP value with 3 decimal places : Risk Factor (RF): mean of SHAP value > 0; Protective Factor (PF): mean of SHAP value < 0; No Effect (NE): mean of SHAP value = 0) by descending were education level (NE), age groups (RF), number of comorbid chronic disease (PF), wealth quantile (NE), marital status (PF), living arrangement (NE), residence (RF), gender (RF) and ethnicity (PF).

Figure 2 The importance sort and mechanism direction of number of comorbid chronic disease with depressive symptoms by using explainable machine learning predictions

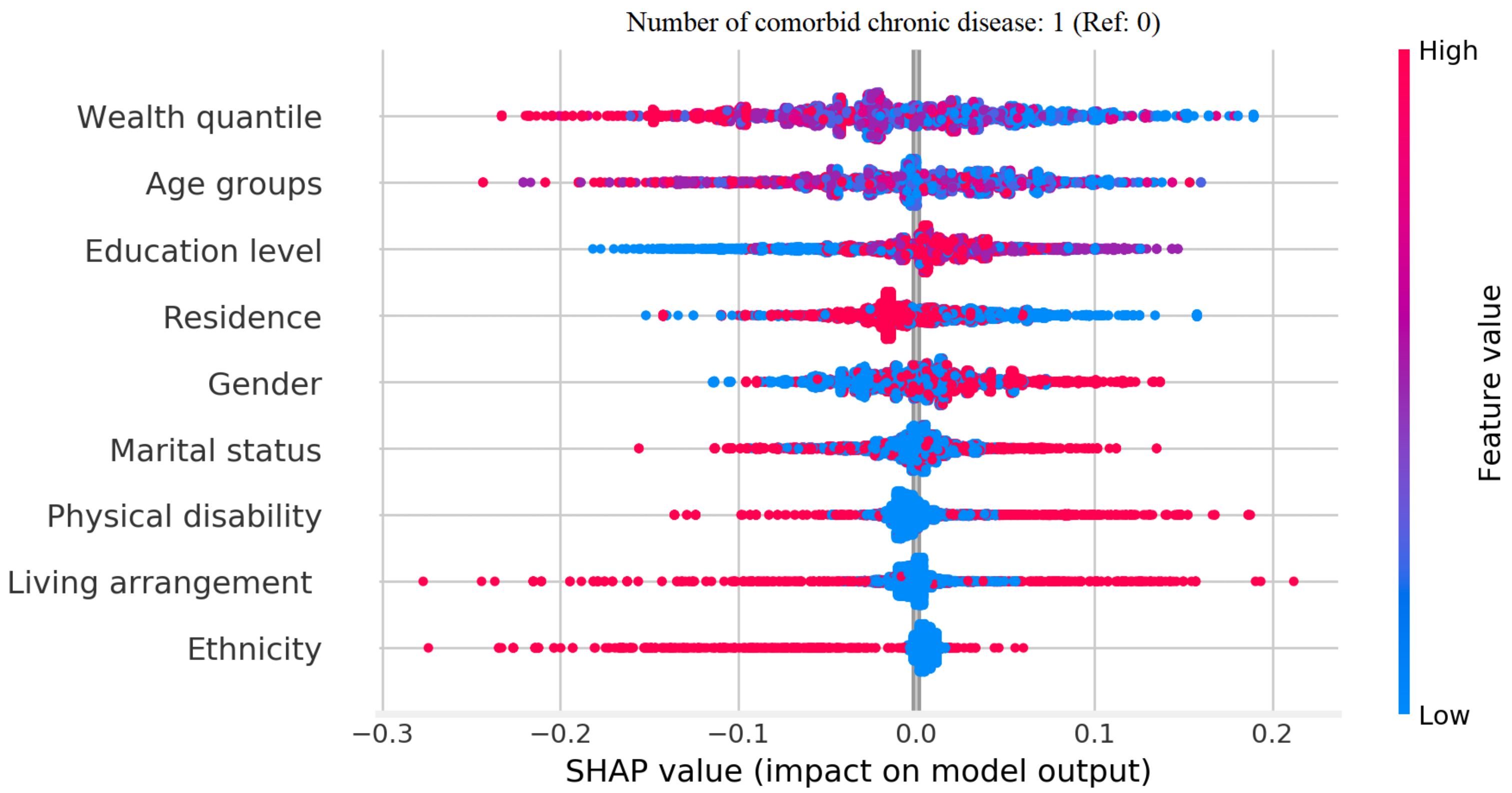

(a) Number of comorbid chronic disease: 1 (Ref: 0)

The importance of variables (Mean of SHAP value with 3 decimal places: Risk Factor (RF): mean of SHAP value > 0; Protective Factor (PF): mean of SHAP value < 0; No Effect (NE): mean of SHAP value = 0) by descending were wealth quantile (NE), age groups (RF), education level (RF), residence (RF), gender (RF), marital status (PF), physical disability (RF), living arrangement (NE) and ethnicity (PR).

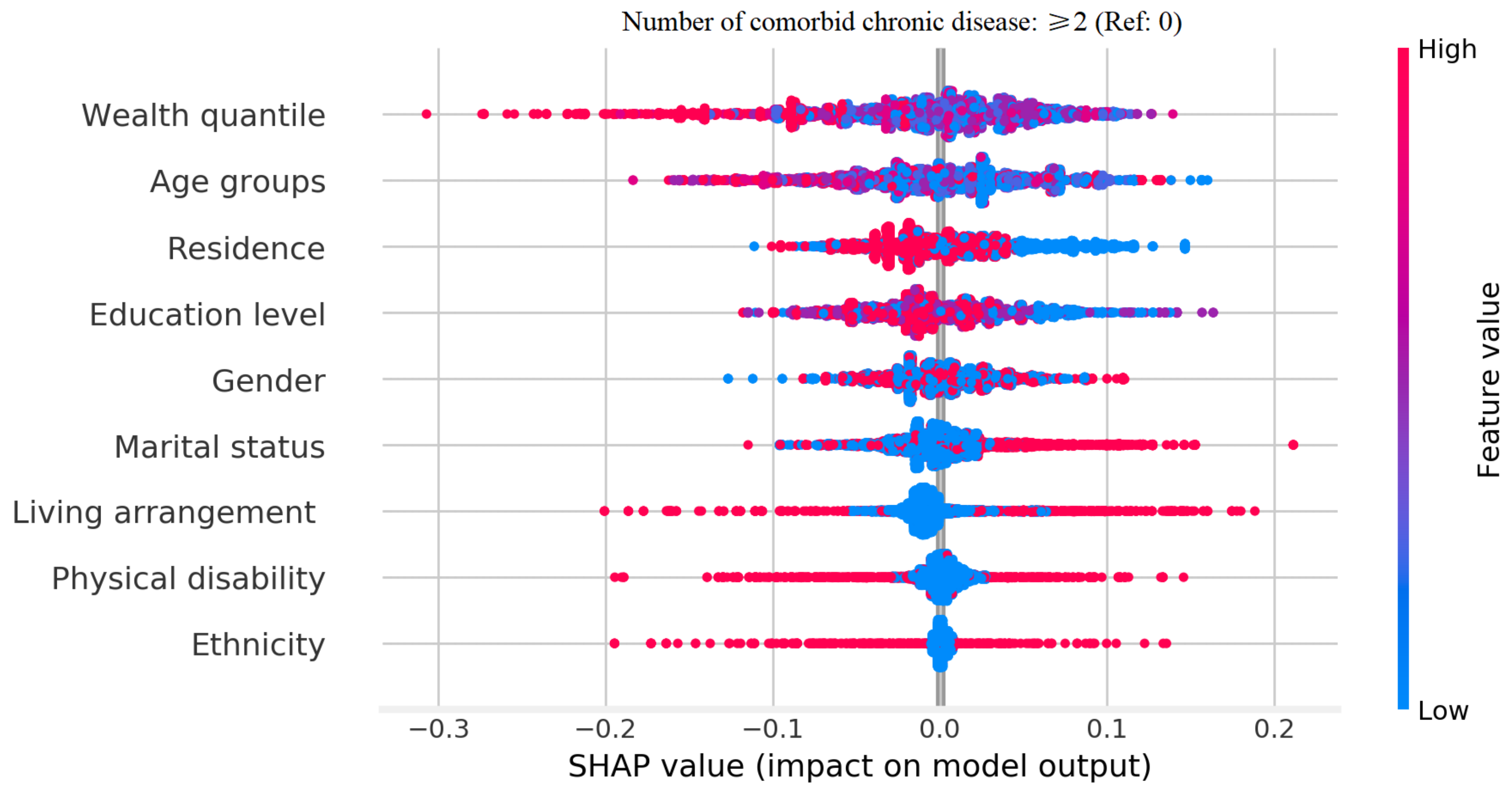

(b) Number of comorbid chronic disease: ⩾2 (Ref: 0)

The importance of variables (Mean of SHAP value with 3 decimal places: Risk Factor (RF): mean of SHAP value > 0; Protective Factor (PF): mean of SHAP value < 0; No Effect (NE): mean of SHAP value = 0) by descending were wealth quantile (PF), age groups (RF), residence (RF), education level (NE), gender (RF), marital status (NF), living arrangement (NE), physical disability (NF) and ethnicity (PF).

Editing Certificate

This document certifies that the manuscript

Estimating causal effects of physical disability and number of comorbid chronic diseases on depressive symptom risk in an elderly Chinese population: A machine learning studyanalysis of cross-sectional baseline data from the China Longitudinal Ageing Social Survey

prepared by the authors

Zhenjie Wang, Hanmo Yang, Chenxi Sun, Shenda Hong

was edited for proper English language, grammar, punctuation, spelling, and overall style by one or more of the highly qualified native English speaking editors at AJE.

This certificate was issued on **March 22, 2023** and may be verified on the AJE website using the verification code **1810-9783-237A-B2F3-A7A3**.

Neither the research content nor the authors' intentions were altered in any way during the editing process. Documents receiving this certification should be English-ready for publication; however, the author has the ability to accept or reject our suggestions and changes. To verify the final AJE edited version, please visit our verification page at aje.com/certificate. If you have any questions or concerns about this edited document, please contact AJE at support@aje.com.

AJE provides a range of editing, translation, and manuscript services for researchers and publishers around the world. For more information about our company, services, and partner discounts, please visit aje.com.

For peer review only - http://bmjopen.bmj.com/site/about/guidelines.xhtml

STROBE Statement—Checklist of items that should be included in reports of ***cross-sectional studies***

	Item No	Recommendation	Page No
**Title and abstract**	1	(*a*) Indicate the study's design with a commonly used term in the title or the abstract	1
		(*b*) Provide in the abstract an informative and balanced summary of what was done and what was found	2-3
**Introduction**			
Background/rationale	2	Explain the scientific background and rationale for the investigation being reported	4
Objectives	3	State specific objectives, including any prespecified hypotheses	4
**Methods**			
Study design	4	Present key elements of study design early in the paper	5
Setting	5	Describe the setting, locations, and relevant dates, including periods of recruitment, exposure, follow-up, and data collection	5
Participants	6	(*a*) Give the eligibility criteria, and the sources and methods of selection of participants	5
Variables	7	Clearly define all outcomes, exposures, predictors, potential confounders, and effect modifiers. Give diagnostic criteria, if applicable	5-6
Data sources/ measurement	8*	For each variable of interest, give sources of data and details of methods of assessment (measurement). Describe comparability of assessment methods if there is more than one group	5-6
Bias	9	Describe any efforts to address potential sources of bias	
Study size	10	Explain how the study size was arrived at	5
Quantitative variables	11	Explain how quantitative variables were handled in the analyses. If applicable, describe which groupings were chosen and why	
Statistical methods	12	(*a*) Describe all statistical methods, including those used to control for confounding	6-8
		(*b*) Describe any methods used to examine subgroups and interactions	6-8
		(*c*) Explain how missing data were addressed	6-8
		(*d*) If applicable, describe analytical methods taking account of sampling strategy	6-8
		(*e*) Describe any sensitivity analyses	6-8
**Results**			
Participants	13*	(a) Report numbers of individuals at each stage of study—eg numbers potentially eligible, examined for eligibility, confirmed eligible, included in the study, completing follow-up, and analysed	8-11
		(b) Give reasons for non-participation at each stage	8-11
		(c) Consider use of a flow diagram	8-11
Descriptive data	14*	(a) Give characteristics of study participants (eg demographic, clinical, social) and information on exposures and potential confounders	8-11
		(b) Indicate number of participants with missing data for each variable of interest	8-11
Outcome data	15*	Report numbers of outcome events or summary measures	8-11
Main results	16	(*a*) Give unadjusted estimates and, if applicable, confounder-adjusted estimates and their precision (eg, 95% confidence interval). Make clear which confounders were adjusted for and why they were included	8-11

For peer review only

		(*b*) Report category boundaries when continuous variables were categorized	8-11
		(*c*) If relevant, consider translating estimates of relative risk into absolute risk for a meaningful time period	8-11
Other analyses	17	Report other analyses done—eg analyses of subgroups and interactions, and sensitivity analyses	8-11
**Discussion**			
Key results	18	Summarise key results with reference to study objectives	11
Limitations	19	Discuss limitations of the study, taking into account sources of potential bias or imprecision. Discuss both direction and magnitude of any potential bias	13
Interpretation	20	Give a cautious overall interpretation of results considering objectives, limitations, multiplicity of analyses, results from similar studies, and other relevant evidence	11-13
Generalisability	21	Discuss the generalisability (external validity) of the study results	11-13
**Other information**			
Funding	22	Give the source of funding and the role of the funders for the present study and, if applicable, for the original study on which the present article is based	

*Give information separately for exposed and unexposed groups.

Note: An Explanation and Elaboration article discusses each checklist item and gives methodological background and published examples of transparent reporting. The STROBE checklist is best used in conjunction with this article (freely available on the Web sites of PLoS Medicine at http://www.plosmedicine.org/, Annals of Internal Medicine at http://www.annals.org/, and Epidemiology at http://www.epidem.com/). Information on the STROBE Initiative is available at www.strobe-statement.org.

For peer review only

BMJ Open

Estimating causal effects of physical disability and number of comorbid chronic diseases on risk of depressive symptoms in an elderly Chinese population: a machine learning analysis of cross-sectional baseline data from the China Longitudinal Ageing Social Survey

Journal:	*BMJ Open*
Manuscript ID	bmjopen-2022-069298.R2
Article Type:	Original research
Date Submitted by the Author:	04-May-2023

Complete List of Authors:	Wang, Zhenjie; Peking University<br>Yang, Hanmo; Harvard University<br>Sun, Chenxi; Peking University; Peking University<br>Hong, Shenda; Peking University; Health Science Center of Peking University

**Primary Subject Heading**:	Health informatics
Secondary Subject Heading:	Health informatics
Keywords:	Health informatics < BIOTECHNOLOGY & BIOINFORMATICS, MENTAL HEALTH, Depression & mood disorders < PSYCHIATRY

SCHOLARONE™
Manuscripts

I, the Submitting Author has the right to grant and does grant on behalf of all authors of the Work (as defined in the below author licence), an exclusive licence and/or a non-exclusive licence for contributions from authors who are: i) UK Crown employees; ii) where BMJ has agreed a CC-BY licence shall apply, and/or iii) in accordance with the terms applicable for US Federal Government officers or employees acting as part of their official duties; on a worldwide, perpetual, irrevocable, royalty-free basis to BMJ Publishing Group Ltd ("BMJ") its licensees and where the relevant Journal is co-owned by BMJ to the co-owners of the Journal, to publish the Work in this journal and any other BMJ products and to exploit all rights, as set out in our licence.

The Submitting Author accepts and understands that any supply made under these terms is made by BMJ to the Submitting Author unless you are acting as an employee on behalf of your employer or a postgraduate student of an affiliated institution which is paying any applicable article publishing charge ("APC") for Open Access articles. Where the Submitting Author wishes to make the Work available on an Open Access basis (and intends to pay the relevant APC), the terms of reuse of such Open Access shall be governed by a Creative Commons licence – details of these licences and which Creative Commons licence will apply to this Work are set out in our licence referred to above.

Other than as permitted in any relevant BMJ Author's Self Archiving Policies, I confirm this Work has not been accepted for publication elsewhere, is not being considered for publication elsewhere and does not duplicate material already published. I confirm all authors consent to publication of this Work and authorise the granting of this licence.

For peer review only

Estimating causal effects of physical disability and number of comorbid chronic diseases on risk of depressive symptoms in an elderly Chinese population: a machine learning analysis of cross-sectional baseline data from the China Longitudinal Ageing Social Survey

Zhenjie Wang,[1*] Hanmo Yang,[4] Chenxi Sun,[5] Shenda Hong[2,3*]

[1] Institute of Population Research, Peking University, Beijing 100871, People's Republic of China

[2] National Institute of Health Data Science, Peking University, Beijing 100191, People's Republic of China

[3] Institute of Medical Technology, Health Science Center of Peking University, Beijing 100191, People's Republic of China

[4] T. H. Chan School of Public Health, Harvard University, Boston 02115, United States of America

[5] School of Intelligence Science and Technology and the Key Laboratory of Machine Perception (Ministry of Education) Peking University, Beijing, People's Republic of China

***Correspondence to:**

Shenda Hong

National Institute of Health Data Science, Peking University, Beijing 100191, People's Republic of China

E-mail: hongshenda@pku.edu.cn

Zhenjie Wang

For peer review only

Institute of Population Research, Peking University, No. 5 Yiheyuan Road, Haidian District, Beijing 100871, People's Republic of China

E-mail: zhenjie.wang@pku.edu.cn

ABSTRACT

Objective This study aimed to explore the causal effects of physical disability and number of comorbid chronic diseases on depressive symptoms in an elderly Chinese population.

Design, setting and analysis Cross-sectional, baseline data were obtained from the China Longitudinal Ageing Social Survey (CLASS), a stratified, multistage, probabilistic sampling survey conducted in 2014 that covers 28 of 31 provincial areas in China. The causal effects of physical disability and number of comorbid chronic diseases on depressive symptoms were analyzed using the conditional average treatment effect (CATE) method of machine learning. The causal effects model's adjustment was made for age, gender, residence, marital status, educational level, ethnicity, wealth quantile, and other factors.

Outcome Assessment of the causal effects of physical disability and number of comorbid chronic diseases on depressive symptoms.

Participants 7496 subjects who were 60 years of age or older and who answered the questions on depressive symptoms and other independent variables of interest in a survey conducted in 2014 were included in this study.

Results Physical disability and number of comorbid chronic diseases had causal effects on depressive symptoms. Among the subjects who had one or more functional limitations, the probability of depressive symptoms increased by 22% (95%CI: 19%-24%). For the subjects

For peer review only

who had one chronic disease and those who had two or more chronic diseases, the possibility of depressive symptoms increased by 13% (95%CI: 10%-15%), and 20% (95%CI: 18%-22%), respectively.

Conclusion: The present study provides evidence that the presence of one or more functional limitations affects the occurrence of depressive symptoms among elderly people. The findings of our study are of value in developing programs that are designed to identify elderly individuals who have physical disabilities or comorbid chronic diseases to provide early intervention.

Keywords: Health informatics, mental health, causal effects, older population, Chinese

Strengths and limitations of this study

- The current study used a large, representative population-based sample covering 28 of 31 provincial areas in China.
- The study estimated causal effects using the conditional average treatment effect (CATE) method.
- We were unable to evaluate the effects of lifestyle factors since information on these factors was not collected in the CLASS.
- The findings of this cross-sectional study established a cause-and-effect relationship based on the use of a machine learning algorithm; these must be considered in further research.

For peer review only

For peer review only

INTRODUCTION

Currently, depressive symptoms (DS) are one of the most prevalent mental disorders worldwide.[1] The characteristics of DS include symptoms of sadness, depressed mood, and loss of interest.[1] The presence of DS is usually associated with low quality of life, cancer, chronic diseases, suicide, and other conditions.[2-5] DS can also place a heavy burden on families, communities and health services worldwide.[6, 7] The prevalence of DS varies widely and ranges from 1% to 16% among middle-aged and elderly populations.[8-10] In China, the prevalence of DS among elderly people has increased rapidly from 3.9% to 17% in the past two decades.[11-13]

Many studies have provided evidence that specific demographic variables are associated with DS risk,[14] although the associations are inconsistent.[10, 13] An inverse or U-shaped association between age and DS has been found in many studies.[15-17] Demographic status and level of education were also found to be associated with DS risk.[18-20] However, some studies reported no association of DS with demographic status or level of education.[14,17,21]

Machine learning (ML) models can handle complex relationships between variables and can capture nonlinearities and interactions that may be missed by traditional statistical models. Currently, researcher can evaluate the treatment effect though the average effect treatment effect.[22] CATE (Conditional Average Treatment Effect) estimation is a specific ML technique used in the context of causal inference. CATE aims to estimate the effect of a treatment on an outcome for a specific subpopulation or individual, rather than the average effect across the entire population. Very few studies have used machine learning to obtain evidence regarding the causal effects of physical disability or the number of comorbid chronic

For peer review only

diseases on DS risk in the elderly Chinese population.

In this study, we used machine learning to explore the conditional average treatment effect (CATE) of physical disability and number of comorbid chronic diseases on depressive symptoms using data from the Chinese Longitudinal Ageing Social Survey (CLASS).

METHODS

Data source

We drew the sample used in the current study from the cross-sectional baseline data the Chinese Longitudinal Ageing Social Survey (CLASS), which was conducted in 2014.[23] The CLASS was collected by the National Survey Research Center, Renmin University. The CLASS used a stratified, multistage, probabilistic sampling method to select a nationally representative sample covering 28 of 31 provincial areas in China. A total of 11,511 older adults were surveyed. In the present study, the sample comprised 7496 subjects 60 years of age or older who answered the questions on depressive symptoms and other independent variables of interest collected in 2014. All the participants were interviewed face-to-face by trained interviewers.

Measurement of variables

Depressive symptoms

Depressive symptoms were assessed using the 9-item Center for Epidemiological Studies Depression Scale (CES-D). This scale includes three items designed to assess positive feelings and two items each designed to assess negative emotions, somatic symptoms, and

For peer review only

sense of marginalization. The 9-item CES-D is reliable and valid for detecting nonpsychotic mental disorders among older Chinese adults.[24] For each item, a score of 0 (rarely or none of the time), 1 (some of the time), or 2 (most of the time) was assigned; thus, the total score ranged from 0 to 18. Since the coding of the positive effect items was reversed, a higher score indicates a higher level of depressive symptoms. In the current study, on the 9-item scale, the total possible score was 18 (9 items multiplied by 2, the highest response). A standardized cutoff score of 4.8 for the 9-item form of the CES-D was established using Kohout's formula.[25] In this study, the internal Cronbach's alpha for the nine items was 0.75.

Physical disabilities

Activities of daily living (ADLs) were assessed using the Barthel Index. For each individual, the self-reported number of comorbid chronic diseases that were based on self-reported previous medical record, including health problems such as hypertension, diabetes, heart disease, renal disease, liver disease, stroke, tuberculosis, arthritis, and respiratory diseases, was categorized as "0", "1" or "≥2".

Confounding variables

Confounding variables, which were significantly associated with depressive symptoms,[26] under consideration were gender (male, female), age, marital status (married, widowed/divorced/unmarried), ethnicity (Han, others), residence (rural, urban), educational level (junior high school and above, primary school, never attended school), and living arrangements (lives alone, lives with others). Income was categorized into five levels using

For peer review only

quintiles of household income (yuan) (Q1: ≤ 3000; Q2: >3000 and ≤ 10000; Q3: >10000 and ≤ 24000; Q4: >24000 and ≤ 36000; Q5: >36000).

Estimation of causal effects

Causal analysis aims to determine the causal relationships between variables (treatments) and outcomes. Causal relationships are essentially different from correlations in that causal relationships mainly solve the problem of "why". In this work, we used Causal ML,an open-source Python software provided by Uber, to perform causal analysis using machine learning algorithms based on recent research.[27] Causal ML provides a standard interface that allows users to estimate the average treatment effect (ATE) based on experimental or observational data.

To estimate the causal relationship between the variable of interest (known as treatment indicator E) and the outcome Y, we performed a two-step analysis: (1) we built a machine learning model and (2) we estimated the ATE based on causal ML. First, we built a random forest classifier (RFC) and used it to predict DS using all the variables except the treatment indicator (denoted X).[28] The RFC contains 100 decision trees and uses the Gini purity index to judge the segmentation criteria of a tree node. The maximum depth of a single tree was set to unlimited so that each tree would be segmented until each leaf node of the tree model contained only one sample. The RFC output was 1 if the individual had DS and 0 otherwise. The RFC model was built using a scikit-learn machine learning framework.[29]

We then set the above trained RFC as the base learner for causal ML and estimated the treatment effect based on the algorithm described herein.[30] The algorithm, which uses a single

For peer review only

base learner from the machine learning model and has the treatment indicator as a feature, operates in two stages. In the first stage, the algorithm estimates the average outcomes after specific treatments using the trained RFC model, where the outcome is calculated as $\mu(x) = E(Y|X = x, E = e)$. In the second stage, the ATE was calculated as $\tau(x) = \mu(x, E = 1) - \mu(x, E = 0)$. We also used SHAPley Additive exPlanations (SHAP), a game theory approach, to explain the output of the causal analysis results modeled by the machine learning models.[31] We analyzed the data using Python 3.7.14 based on the anaconda3 development platform, causalml version 0.10.0, sklearn version 0.23.2, pandas version 1.0.1, and numpy version 1.21.0.

Survey conduct and consent

The interviewer-administered questionnaire was prepared in the local language (Chinese). Consent to participation in the study was received from each participant prior to data collection, and the data collectors were trained to provide any requested information or clarification at any time during the interview. Only participants who wished to continue participating in the study after providing informed consent were included in the study.

Patient and public involvement

None.

RESULTS

For peer review only

Descriptive statistics

The characteristics of subjects with and without depressive symptoms (DS) are summarized in Table 1. The overall average Center for Epidemiological Studies Depression Scale (CES-D) score was 4.56 (standard deviation: 3.56). The prevalence of DS in the Chinese population over 60 years of age was 43%. In the current study, male subjects, urban residents, people living with others, and people of Han ethnicity accounted for the majority of elderly Chinese individuals with DS.

Table 1. Subjects' characteristics by their depressive symptom status according to the 9-item Center for Epidemiological Studies Depression Scale (CES-D) among the elderly population in China

Characteristics	Lower than depressive symptom cut-off point*	Higher than depressive symptom cut-off point*	*P*
Age (years)			
60-64	1603 (37.2)	1068 (33.5)	< 0.001
65-69	988 (22.9)	704 (22.1)	
70-74	766 (17.8)	547 (17.2)	
75-79	530 (12.3)	471 (14.8)	
80+	422 (9.8)	397 (12.5)	
Residence			
Rural	1207 (28.0)	1315 (41.3)	< 0.001
Urban	3102 (72.0)	1872 (58.8)	
Gender			
Male	2427 (56.3)	1627 (51.1)	< 0.001
Female	1882 (43.7)	1563 (48.9)	
Marital status			
Married	3316 (77.0)	2038 (63.9)	< 0.001
Widowed/divorced/unmarried	993 (23.0)	1149 (36.1)	
Educational level			
Never attended school	651 (15.1)	816 (25.6)	< 0.001
Primary school	1409 (32.7)	1266 (39.7)	
Junior high school and above	2249 (52.2)	1105 (34.7)	
Ethnicity			
Han	4074 (94.5)	2990 (93.8)	0.19
Other	235 (5.5)	197 (6.2)	
Living arrangement			
Live with others	3925 (91.1)	2666 (83.7)	< 0.001

For peer review only

Live alone	384 (8.9)	521 (16.3)	
Physical disability			
No functional limitations	3988 (92.6)	2531 (79.4)	< 0.001
One or more functional limitations	321 (7.4)	656 (20.6)	
Wealth quantile			
Q1 (lowest)	691 (16.0)	925 (29.0)	< 0.001
Q2	694 (16.1)	717 (22.5)	
Q3	1066 (24.7)	705 (22.1)	
Q4	1002 (23.3)	520 (16.3)	
Q5 (highest)	856 (19.9)	320 (10.1)	
Number of comorbid chronic diseases			
0	1463 (34.0)	551 (17.3)	< 0.001
1	1307 (30.3)	917 (28.8)	
≥2	1539 (35.7)	1719 (53.9)	

* For the current study, on a 9-item scale, the total possible score was 18 (9 items multiplied by 2, the highest response). The standardized cut-off point was 4.8 by using Kohout's formula.

Estimated causal effects

The estimated causal effects of physical disability and chronic diseases on depressive symptoms are presented in Table 2. Physical disability and the number of comorbid chronic diseases had causal effects on DS. Among the subjects who had one or more functional limitations, those who had one chronic disease, and those who had two or more chronic diseases, the risk probability of DS increased by 22%, 13%, and 20%, respectively.

Table 2. Estimated causal effects of physical disability and chronic disease on depressive symptoms

	Reference		Causal effect (95% CI)
Physical disability *	No functional problems	One or more functional limitations	0.22 (0.19, 0.24)
Number of comorbid chronic diseases†	0	1	0.13 (0.10, 0.15)
		≥2	0.20 (0.18, 0.22)

*Taking into consideration the causal effects of age, gender, residence, marital status, educational level, ethnicity, wealth quantile, and number of comorbid chronic diseases.

†Taking into consideration the causal effects of age, gender, residence, marital status, educational level, ethnicity, wealth quantile, and physical disability.

For peer review only

The effects of the mechanism direction of physical disability and number of comorbid chronic diseases on DS are presented in Figures 1-2 in descending order of importance In descending order of importance among subjects who had one or more functional limitations, the variables are as follows: educational level, age, number of comorbid chronic diseases, wealth quantile, marital status, living arrangement, residence, gender and ethnicity. In descending order of importance among subjects who had only one chronic disease, the variables are as follows: wealth quantile, age, educational level, residence, gender, marital status, physical disability, living arrangement and ethnicity. In descending order of importance among subjects who had one or more functional limitations, the variables are as follows: wealth quantile, age, residence, educational level, gender, marital status, living arrangement, physical disability and ethnicity. Age, residence, gender, and physical disability contributed to DS risk among subjects who had one or more chronic diseases.

DISCUSSION

Overall, physical disability and the number of comorbid chronic diseases present had causal effects on depressive symptom (DS) risk. Subjects who had one or more functional limitations had a 22% increased risk of DS. Subjects who had one chronic disease and those who had two or more chronic diseases showed increases of 13% and 20%, respectively, in their DS risk.

In the current study, we found causal effects of physical disability and comorbid chronic diseases on DS. A previous study suggested that some health variables, such as poor self-rated health and disability, are associated with DS, indicating that health status tends to have similar effects on mental health regardless of social context.[32] Physical disability is considered an inability or a diminished capacity to perform basic self-care activities. Physical disability lessens an individual's ability to interact with the physical and social environment, hence

For peer review only

increasing the risk of DS.[33] Increased disability in activities of daily living (ADLs) and instrumental activities of daily living (IADLs) have been proven to be connected with depressive symptoms.[34,35] Failure to perform ADLs has been positively associated with depressive symptoms in elderly people, and physical disability accounts for incidences of depressive symptoms in elderly individuals.[36,37] Furthermore, the finding that physical disability leads to an increase in depressive symptoms in geriatric individuals over time is supported by longitudinal studies.[38]Given the influence of physical disability and depressive symptoms on the well-being of elderly individuals, it is urgent to find ways to address these issues. The results presented here demonstrate that impairment of ADLs has a significant effect on depressive symptoms in elderly people, consistent with the findings of previous studies that showed a relationship between limitations on activity and psychosocial problems.[33,34]

The number of chronic diseases present in an individual was found to be associated with DS risk, consistent with previous studies. These associations could be explained by several factors, including unhealthy lifestyle, shared genetic influences, and inflammation related to disease.[39] Previous studies have suggested that experiencing pain and physical disability could affect mood in elderly individuals.[20] Another explanation is that subjects with depressive symptoms might be more sedentary than those without depressive symptoms; such a lifestyle could cause increased bone resorption and decreased bone formation.[40,41]

Causal inference using machine learning methods such as CATE (Conditional Average Treatment Effect) can be a powerful tool for analyzing observational data. They model the treatment effect using regression or decision trees. Machine learning can learn causal effects

For peer review only

because it can identify and account for confounding factors, which are variables that may affect both the treatment and outcome variables. By controlling for confounding factors, machine learning algorithms can estimate the causal effect of the treatment variable on the outcome variable, even in the absence of a randomized controlled trial.Specifically, CATE aims to estimate the effect of a treatment on an outcome for a specific subpopulation or individual, rather than the average effect across the entire population. This method can help identify treatment effects for different subgroups of the population and enable personalized treatment recommendations.

Strengths and limitations

Our study has many strengths, including a large sample size, a population-based design, and adjustment for a wide range of socioeconomic characteristics. Another noticeable strength is that the measurement of all physical illnesses took place prior to the CES-D measurement, minimizing the risk of reverse causation. However, our study also has several limitations that should be taken into account by future researchers. First, the physical conditions information were self-reported, which should also be considered for future research. Moreover, although we used machine learning to estimate the causal effects of the factors on DS, these findings should be interpreted cautiously in view of the cross-sectional nature of the data used in the present analysis. We will be able to extend the current study to determine causality when longitudinal data are available. Although the current data were collected in 2014, they are available on the web. Because all the information was collected by trained interviewers, the quality of the survey was reliable. This point should also be considered with caution by future

For peer review only

researchers. It is important to note that the CLASS does not provide information on lifestyle factors (i.e., weight, height, smoking, alcohol consumption, and other factors), some of which have been suggested as risk factors for depression.[42-44]

CONCLUSION

In conclusion, in this large population-based study in an elderly Chinese population, physical disability and the number of comorbid chronic diseases were found to increase DS risk. The present study provides evidence of the effect of one or more functional limitations on the occurrence of depressive symptoms among elderly people. This is helpful in planning preventive measures and in improving knowledge of how to treat depressive symptoms. The findings made in our study are valuable for the development of prevention programs designed to identify elderly individuals who have physical disabilities or a number of comorbid chronic diseases and provide early intervention.

Acknowledgments We would like to thank the Institute of Gerontology and National Survey Research Center at Renmin University of China for providing the CLASS data.

Contributors Conceptualization: Z. W.; Methodology: S. H.; Software: Z. W. and S. H.; Writing: Z. W.; Writing—review: Z. W., S. H., H. Y. and C. S.; Writing—editing: H. Y. and C. S.

Funding This work was supported by the National Social Science Fund of China (No. 20BRK020), the National Natural Science Foundation of China (No. 62102008) and the

For peer review only

Economic & Social Research Council (ESRC) (No. ES/P011055/1). The funding agencies did not participate in the research or review any details of this study, and the authors are independent of the funders.

Competing interests None declared.

Patient consent for publication Not needed.

Ethics approval All procedures performed in this study involving human participants were conducted in accordance with the ethical standards of the institutional and/or national research committee and with the 1964 Helsinki Declaration and its later amendments or comparable ethical standards. The surveys were conducted within the legal framework governed by statistical law in China. This survey was approved by Renmin University of China. Our group received permission for use of the CLASS data from the National Survey Research Center at Renmin University of China.

Provenance and peer review Not commissioned; externally peer reviewed.

Data availability statement The datasets generated and/or analyzed during the current study are available at the China Longitudinal Ageing Social Survey (CLASS) repository (http://class.ruc.edu.cn/index.php?r=index/index&hl=en).

REFERENCES

1. Whiteford HA, Ferrari AJ, Degenhardt L, *et al*. Global Burden of Mental, Neurological, and Substance Use Disorders: An Analysis from the Global Burden of Disease Study 2010. *PLoS One*. 2015;10:e0116820.

2. Dalton SO, Laursen TM, Ross L, *et al*. Risk for hospitalization with depression after a

For peer review only

cancer diagnosis: a nationwide, population-based study of cancer patients in Denmark from 1973 to 2003. *J Clin Oncol.* 2009; 27:1440–1445.

3. Hsu YT, Liao CC, Chang SN, Y *et al*. Increased Risk of Depression in Patients with Parkinson Disease: A Nationwide Cohort Study. *Am J Geriatr Psychiatry.* 2015; 23:934–940.
4. Ayerbe L, Ayis S, Wolfe CD, *et al*. Natural history, Predictors and outcomes of depression after stroke: systematic review and meta-analysis. *Br J Psychiatry.* 2013; 202:14-21.
5. Cuijpers P, Vogelzangs N, Twisk J, *et al*. Comprehensive meta-analysis of excess mortality in depression in the general community versus patients with specific illnesses. *Am J Psychiatry.* 2014; 171: 453–462.
6. World Health Organization. Depression–a global public health concern. 2012, http://hesp-news.org/2012/10/05/depression-a-global public-health-concern/ (accessed: 2018-11-29).
7. Walker ER, McGee RE, Druss BG. Mortality in mental disorders and global disease burden implications: a systematic review and meta-analysis. *JAMA Psychiatry.* 2015; 72:334–341.
8. Wilhelm K, Mitchell P, Slade T, *et al*. Prevalence and correlates of DSM-IV major depression in an Australian national survey. *J Affect Disord.* 2003; 75:155–162.
9. Waraich P, Goldner E M, Somers J M, *et al*. Prevalence and incidence studies of mood disorders: a systematic review of the literature. *Can J Psychiatry*. 2004; 49:124–138.
10. Djernes JK. Prevalence and predictors of depression in populations of elderly: a review. *Acta Psychiatr Scand.* 2006;113:372–387.

For peer review only

11. Chen R, Copeland J R, Wei L. A meta-analysis of epidemiological studies in depression of older people in the People's Republic of China. *Int J Geriatr Psychiatry.* 1999; 14:821–830.

12. Li D, Zhang DJ, Shao JJ, *et al*. A meta-analysis of the prevalence of depressive symptoms in Chinese older adults. *Arch Gerontol Geriatr*. 2014; 58:1–9.

13. Zhang L, Xu Y, Nie H, *et al*. The prevalence of depressive symptoms among the older in China: a meta-analysis. *Int J Geriatr Psychiatry.* 2012; 27:900–906.

14. Gao S, Jin Y, Unverzagt FW, *et al*. Correlates of depressive symptoms in rural elderly Chinese. *Int J Geriatr Psychiatry.* 2009; 24:1358–1366.

15. Kessler RC, Birnbaum HG, Shahly V, *et al*. Age differences in the prevalence and co-morbidity of DSM-IV major depressive episodes: results from the WHO World Mental Health Survey Initiative. *Depress Anxiety.* 2010; 27:351–364.

16. Pan A, Franco OH, Wang YF, *et al*. Prevalence and geographic disparity of depressive symptoms among middle-aged and elderly in China. *J Affect Disord.* 2008;105:167–175.

17. Yunming L, Changsheng C, Haibo T, *et al*. Prevalence and risk factors for depression in older people in Xi'an China: a community-based study. *Int J Geriatr Psychiatry.* 2012; 27(1):31-9.

18. Lorant V, Croux C, Weich S, *et al*. Depression and socio-economic risk factors: 7-year longitudinal population study. *Br J Psychiatry*. 2007; 190:293–298.

19. Van d V, Bracke P, Levecque K. Gender differences in depression in 23 European countries. Cross-national variation in the gender gap in depression. *Soc Sci Med*. 2010; 71:305–313.

For peer review only

20. Zhou X, Bi B, Zheng L, *et al*. The prevalence and risk factors for depression symptoms in a rural Chinese sample population. *PLoS One.* 2014; 9:e99692.

21. van der Wurff F B, Beekman A T, *et al*. Prevalence and risk-factors for depression in elderly Turkish and Moroccan migrants in the Netherlands. *J Affect Disord.* 2004; 83:33–41.

22. Pearl, J. Causality: Models, Reasoning, and Inference. Cambridge University Press, Cambridge, 2009.

23. The Chinese Longitudinal Ageing Social study. Available: http://class.ruc.edu.cn/English/Home.htm

24. Silverstein M, Cong Z, Li S. Intergenerational transfers and living arrangements of older people in rural China: consequences for psychological wellbeing. *J. Gerontol*. 2006; 61:S256–S266.

25. Kohout FJ, Berkman LF, Evans DA, Cornoni-Huntley J. Two shorter forms of the CES-D depression symptoms index. *J Aging Health.* 1993;5(2):179–193.

26. Wang Z, Yang H, Guo Z, Liu B, Geng S. Socio-demographic characteristics and co-occurrence of depressive symptoms with chronic diseases among older adults in China: the China longitudinal ageing social survey. *BMC Psychiatry*. 2019;19(1):310.

27. Chen H, Harinen T, Lee JY, *et al*. Causalml: python package for causal machine learning. *arXiv.* (2020).

28. Breiman L ."Random Forests." *Machine Learning*. 2001; 45(1), 5-32.

For peer review only

29. Pedregosa F, Varoquaux G, Gramfort A, *et al*. Scikit-learn: machine learning in python. Scikit-learn: Machine learning in Python. *Journal of machine Learning research*. 2012;12: 2825-2830.

30. Künzel Sren R, Sekhon JS, Bickel PJ, Yu B. Meta-learners for estimating heterogeneous treatment effects using machine learning. *Proc Natl Acad Sci U S A*. 2017; 116: 4156-4165.

31. Lundberg S, Lee SI. A Unified Approach to Interpreting Model Predictions. In Proceedings of the 31st international conference on neural information processing system. 2017; 4768-4777.

32. Kino S, Jang SN, Kawachi I. Healthy but unhappy? Cross-national comparative analysis of depressive symptoms in Japanese vs. Korean elders. *Arch Gerontol Geriatr.* 2021; 95:104426.

33. Bruce ML. Depression and disability in late life: directions for future research. *Am J Geriatr Psychiatry*. 2001;.9(2):102-12.

34. Bowen ME, Ruch A. Depressive Symptoms and Disability Risk Among Older White and Latino Adults by Nativity Status. *J Aging Health*. 2015; 27(7):1286-305.

35. Hybels CF, Pieper CF, Blazer DG. The complex relationship between depressive symptoms and functional limitations in community-dwelling older adults: the impact of subthreshold depression. *Psychol Med*. 2009; 39(10):1677-88.

36. Newsom JT, Schulz R. Social support as a mediator in the relation between functional status and quality of life in older adults. *Psychol Aging*. 1996; 11(1):34-44.

37. Kennedy GJ, Kelman HR, Thomas C. The emergence of depressive symptoms in late life:

For peer review only

the importance of declining health and increasing disability. *J Community Health*. 1990; 15(2):93-104.

38. Lin IF, Wu HS. Does informal care attenuate the cycle of ADL/IADL disability and depressive symptoms in late life? *J Gerontol B Psychol Sci Soc Sci.* 2011; 66(5):585-94.

39. Jia LN, Zhuang HL, Wang XY, *et al*. Depression status of the elderly and its relationship with quality of life in community of Fuzhou city. *Chinese Journal of Geriatrics*. 2011; 30(11):970–973. (Chinese)

40. Bonanno GA, Westphal M, Mancini AD. Resilience in the face of loss and potential trauma. *Annu Rev Clin Psychol.* 2011; 7:511-35.

41. Assil S, Zeidan Z. Prevalence of depression and associated factors among elderly Sudanese: a household survey in Khartoum State. *East Mediterr Health J*. 2013; 19(5):435.

42. .Wiltink J, Michal M, Wild PS, et al. Associations between depression and different measures of obesity (BMI, WC, WHtR, WHR). *BMC Psychiatry*. 2013;13:223.

43. Lopresti AL, Hood SD, Drummond PD. A review of lifestyle factors that contribute to important pathways associated with major depression: diet, sleep, and exercise. *J Affect Disord*. 2013;148:12–27.43.

44. Lo A. Lifestyle factors on depression. In: Pachana N, editor. Encyclopedia of Geropsychology. Springer (2015)

Figure legends

For peer review only

Figure 1. The importance sort and mechanism direction of physical disability with depressive symptoms by using explainable machine learning predictions

Figure 2. The importance sort and mechanism direction of number of comorbid chronic disease with depressive symptoms by using explainable machine learning predictions

For peer review only

Figure 1 The importance sort and mechanism direction of physical disability with depressive symptoms by using explainable machine learning predictions

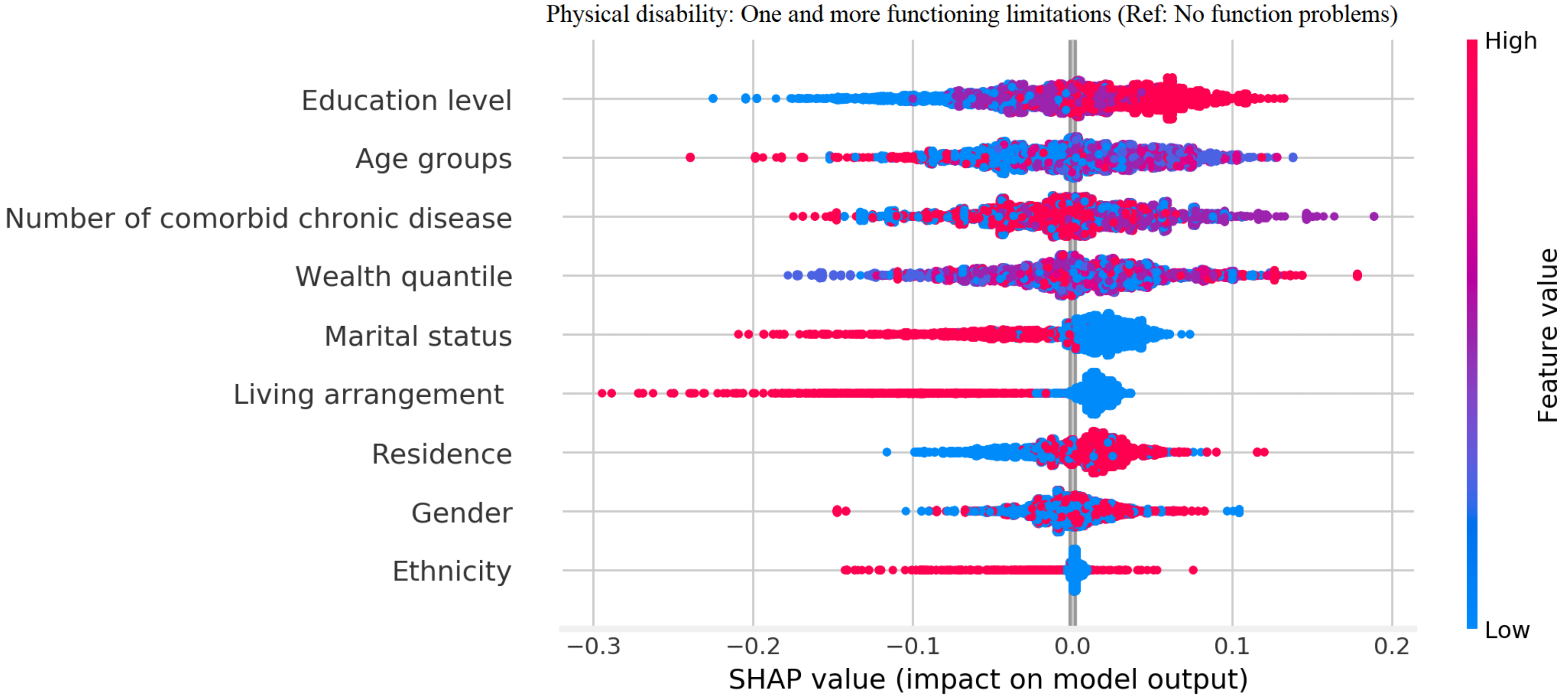

The importance of variables (Mean of SHAP value with 3 decimal places : Risk Factor (RF): mean of SHAP value > 0; Protective Factor (PF): mean of SHAP value < 0; No Effect (NE): mean of SHAP value = 0) by descending were education level (NE), age groups (RF), number of comorbid chronic disease (PF), wealth quantile (NE), marital status (PF), living arrangement (NE), residence (RF), gender (RF) and ethnicity (PF).

Figure 2 The importance sort and mechanism direction of number of comorbid chronic disease with depressive symptoms by using explainable machine learning predictions

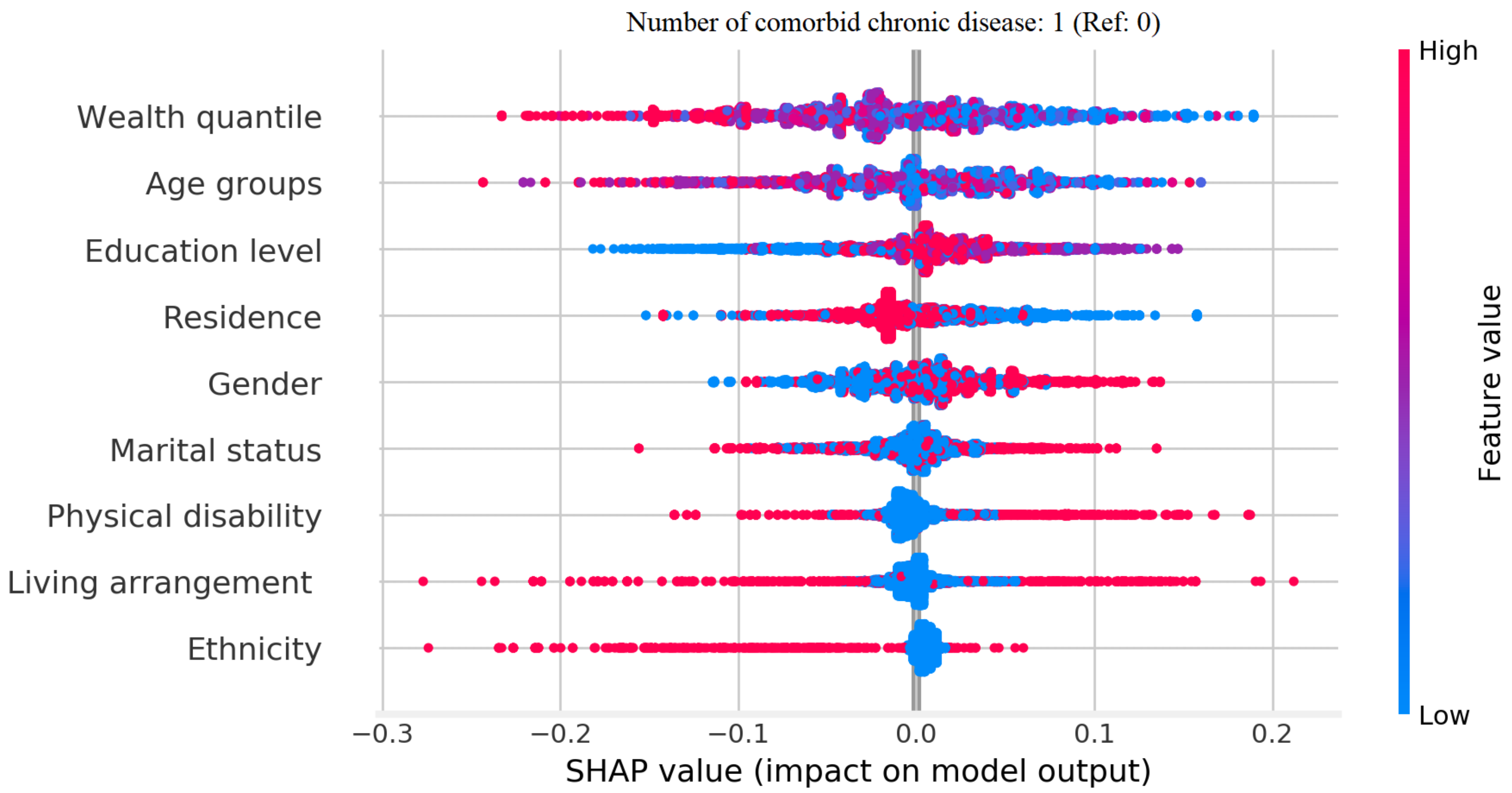

(a) Number of comorbid chronic disease: 1 (Ref: 0)

The importance of variables (Mean of SHAP value with 3 decimal places: Risk Factor (RF): mean of SHAP value > 0; Protective Factor (PF): mean of SHAP value < 0; No Effect (NE): mean of SHAP value = 0) by descending were wealth quantile (NE), age groups (RF), education level (RF), residence (RF), gender (RF), marital status (PF), physical disability (RF), living arrangement (NE) and ethnicity (PR).

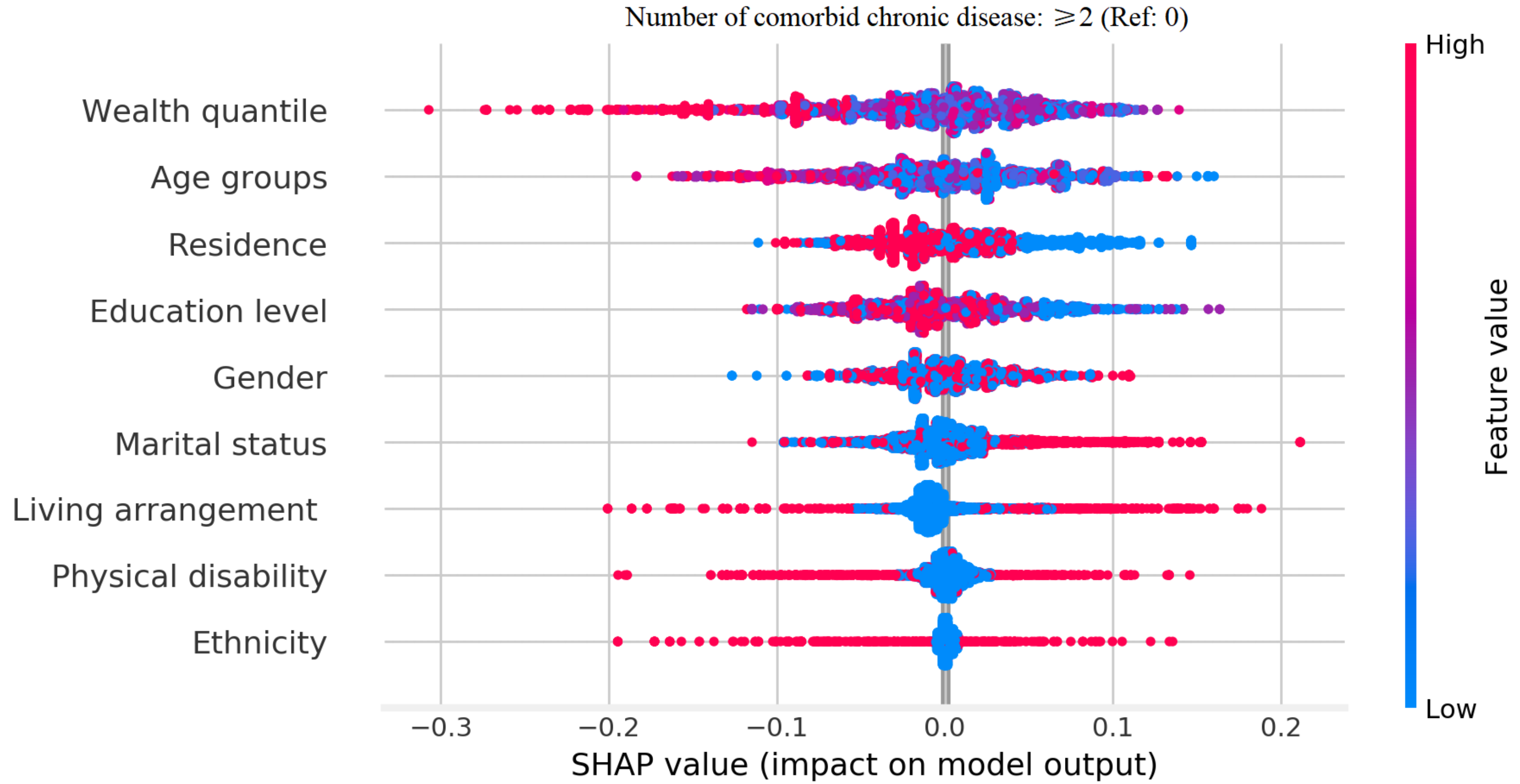

(b) Number of comorbid chronic disease: ⩾2 (Ref: 0)

The importance of variables (Mean of SHAP value with 3 decimal places: Risk Factor (RF): mean of SHAP value > 0; Protective Factor (PF): mean of SHAP value < 0; No Effect (NE): mean of SHAP value = 0) by descending were wealth quantile (PF), age groups (RF), residence (RF), education level (NE), gender (RF), marital status (NF), living arrangement (NE), physical disability (NF) and ethnicity (PF).

STROBE Statement—Checklist of items that should be included in reports of ***cross-sectional studies***

	Item No	Recommendation	Page No
**Title and abstract**	1	(*a*) Indicate the study's design with a commonly used term in the title or the abstract	1
		(*b*) Provide in the abstract an informative and balanced summary of what was done and what was found	2-3
**Introduction**			
Background/rationale	2	Explain the scientific background and rationale for the investigation being reported	4
Objectives	3	State specific objectives, including any prespecified hypotheses	4
**Methods**			
Study design	4	Present key elements of study design early in the paper	5
Setting	5	Describe the setting, locations, and relevant dates, including periods of recruitment, exposure, follow-up, and data collection	5
Participants	6	(*a*) Give the eligibility criteria, and the sources and methods of selection of participants	5
Variables	7	Clearly define all outcomes, exposures, predictors, potential confounders, and effect modifiers. Give diagnostic criteria, if applicable	5-6
Data sources/ measurement	8*	For each variable of interest, give sources of data and details of methods of assessment (measurement). Describe comparability of assessment methods if there is more than one group	5-6
Bias	9	Describe any efforts to address potential sources of bias	
Study size	10	Explain how the study size was arrived at	5
Quantitative variables	11	Explain how quantitative variables were handled in the analyses. If applicable, describe which groupings were chosen and why	
Statistical methods	12	(*a*) Describe all statistical methods, including those used to control for confounding	6-8
		(*b*) Describe any methods used to examine subgroups and interactions	6-8
		(*c*) Explain how missing data were addressed	6-8
		(*d*) If applicable, describe analytical methods taking account of sampling strategy	6-8
		(*e*) Describe any sensitivity analyses	6-8
**Results**			
Participants	13*	(a) Report numbers of individuals at each stage of study—eg numbers potentially eligible, examined for eligibility, confirmed eligible, included in the study, completing follow-up, and analysed	8-11
		(b) Give reasons for non-participation at each stage	8-11
		(c) Consider use of a flow diagram	8-11
Descriptive data	14*	(a) Give characteristics of study participants (eg demographic, clinical, social) and information on exposures and potential confounders	8-11
		(b) Indicate number of participants with missing data for each variable of interest	8-11
Outcome data	15*	Report numbers of outcome events or summary measures	8-11
Main results	16	(*a*) Give unadjusted estimates and, if applicable, confounder-adjusted estimates and their precision (eg, 95% confidence interval). Make clear which confounders were adjusted for and why they were included	8-11

For peer review only

For peer review only - http://bmjopen.bmj.com/site/about/guidelines.xhtml

		(*b*) Report category boundaries when continuous variables were categorized	8-11
		(*c*) If relevant, consider translating estimates of relative risk into absolute risk for a meaningful time period	8-11
Other analyses	17	Report other analyses done—eg analyses of subgroups and interactions, and sensitivity analyses	8-11
**Discussion**			
Key results	18	Summarise key results with reference to study objectives	11
Limitations	19	Discuss limitations of the study, taking into account sources of potential bias or imprecision. Discuss both direction and magnitude of any potential bias	13
Interpretation	20	Give a cautious overall interpretation of results considering objectives, limitations, multiplicity of analyses, results from similar studies, and other relevant evidence	11-13
Generalisability	21	Discuss the generalisability (external validity) of the study results	11-13
**Other information**			
Funding	22	Give the source of funding and the role of the funders for the present study and, if applicable, for the original study on which the present article is based	

*Give information separately for exposed and unexposed groups.

Note: An Explanation and Elaboration article discusses each checklist item and gives methodological background and published examples of transparent reporting. The STROBE checklist is best used in conjunction with this article (freely available on the Web sites of PLoS Medicine at http://www.plosmedicine.org/, Annals of Internal Medicine at http://www.annals.org/, and Epidemiology at http://www.epidem.com/). Information on the STROBE Initiative is available at www.strobe-statement.org.

For peer review only
